# Rhenium(V) Complexes as Cysteine-Targeting Coordinate Covalent Warheads

**DOI:** 10.1021/acs.jmedchem.2c02074

**Published:** 2023-02-08

**Authors:** Johannes Karges, Seth M. Cohen

**Affiliations:** Department of Chemistry and Biochemistry, University of California, San Diego, La Jolla, California 92093, United States

## Abstract

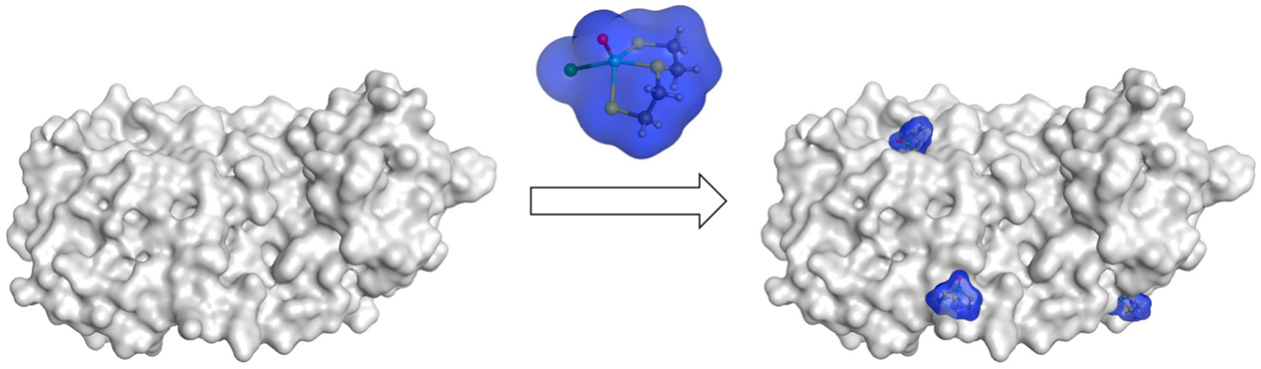

Interest in covalent enzyme inhibitors as therapeutic agents has seen a recent resurgence. Covalent enzyme inhibitors typically possess an organic functional group that reacts with a key feature of the target enzyme, often a nucleophilic cysteine residue. Herein, the application of small, modular Re^V^ complexes as inorganic cysteine-targeting warheads is described. These metal complexes were found to react with cysteine residues rapidly and selectively. To demonstrate the utility of these Re^V^ complexes, their reactivity with SARS-CoV-2-associated cysteine proteases is presented, including the SARS-CoV-2 main protease and papain-like protease and human enzymes cathepsin B and L. As all of these proteins are cysteine proteases, these enzymes were found to be inhibited by the Re^V^ complexes through the formation of adducts. These findings suggest that these Re^V^ complexes could be used as a new class of warheads for targeting surface accessible cysteine residues in disease-relevant target proteins.

## Introduction

**Figure 1:**
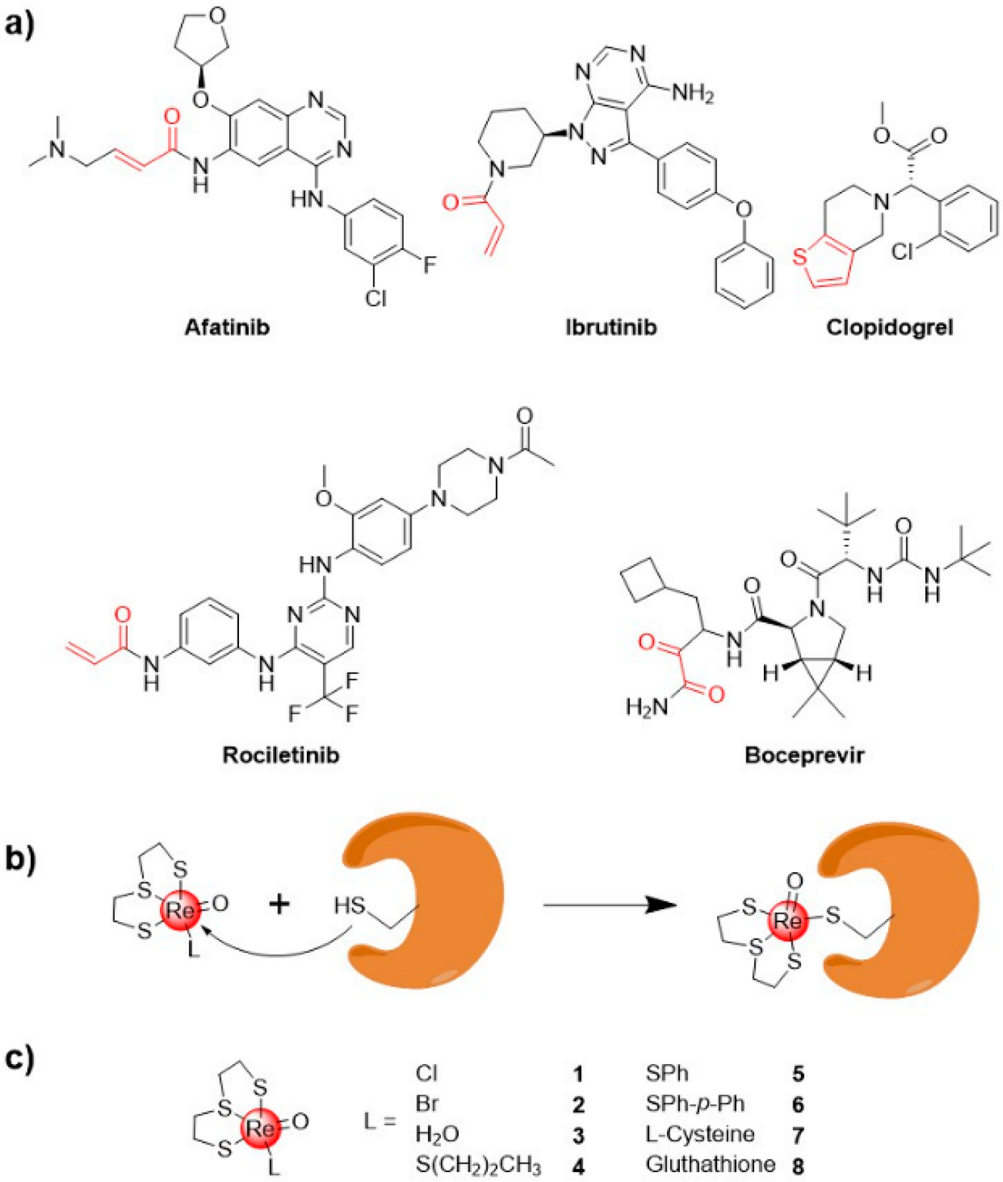
(a) Selected examples of approved covalent drugs with their respective warhead colored red. (b) Concept of a Re(2,2′-thiodiethanethiolate)(L) complex (L = labile ligand) as a coordinate covalent warhead. (c) Chemical structures of metal complexes investigated in this study.

Over the past century, covalent drugs have had a tremendous impact on the quality of human life. Some of the most famous examples include aspirin, which covalently modifies cyclooxygenase by acetylation of a serine residue in the proximity of the active site,^1^ and penicillin, which covalently binds an active site serine residue on the penicillin binding protein, inhibiting a key step in the synthesis of bacterial cell walls.^2^ Despite their clinical success, there has been some reluctance to develop covalent inhibitors due to concerns regarding their off-target reactivity and potential side effects.^3,4^ However, there has been a recent renewal of interest in covalent inhibitors.^5,6^ Covalent inhibitors typically have an organic functional group that reacts with a specific amino acid residue on the target protein. This reactive functional group is also termed a chemical “warhead”. Based on the high reactivity and low occurrence in the human proteome, a majority of covalent drugs target cysteine residues.^7,8^ Compounds such as afatinib, ibrutinib, rociletinib, clopidogrel, and boceprevir (Figure 1a) have been clinically approved as covalent drugs for the treatment of heart diseases, stroke, hepatitis C, and various cancers (including non-small cell lung carcinoma, mantle cell lymphoma, and others).^9^

To date, the majority of covalent warheads have been based on acrylamides or α,β-unsaturated carbonyl moieties (reactive warheads are colored red in Figure 1a). Despite their clinical success, the use of these organic warheads can be limited due to the slow reactivity with target residues or inability to perform nucleophilic attack due to steric hindrance of the warhead or limited space within the active site.^10,11^ To overcome these limitations, many efforts have focused on the development of novel warheads. Examples of newly developed cysteine-targeting warheads include, but are not limited to, alkenyl- or alkynyl-substituted heteroarenes,^12^ alkyl halides,^13^ epoxides and other three-membered heterocycles,^14^ moieties for nucleophilic aromatic substitution reactions,^15^ and moieties that react via release of molecular strain.^16^ Despite these advancements, these moieties have yet to be employed in clinically approved therapeutics.

Beyond organic compounds, metal complexes are receiving increasing interest as potential enzyme inhibitors. Metal complexes can release labile ligands, resulting in an open coordination site that can interact with biomolecules generating coordinate covalent adducts, which results in desired pharmacological outcomes.^17^ Among these studies, Fricker and co-workers have screened various metal complexes as enzyme inhibitors for the treatment of parasitic diseases. In particular, Re^V^ complexes were found to be highly potent cysteine protease inhibitors.^18−20^ These studies were focused on the development of a potent inhibitor for cathepsin B and cathepsin K. The most active compounds were counterscreened for activity against the parasitic cysteine proteases cruzain from *Trypanosoma cruzi*, cpB from *Leishmania major*, and chymotrypsin and found to inhibit the growth of *T. cruzi* parasites. The authors of this study hypothesized that these compounds could form coordinate covalent adducts with the target enzymes. However, studies of the reactivity, verification of metal complex–enzyme adduct formation, or the targeted amino acid residue of the enzymes were not described. Importantly, studies of the potential use of Re^V^ complexes in biomedical applications have indicated that these types of complexes demonstrate low cytotoxicity in mammalian cells.^18,21,22^

Herein, the synthesis and in-depth biochemical investigation of small, modular Re^V^ complexes as inorganic cysteine-targeting binders is proposed. The metal complexes were designed with a sterically nondemanding but tightly bound tridentate S,S,S spectator ligand and a sterically tunable but labile monodentate labile ligand that is released upon protein binding (Figure 1b). The reactivity of the complexes toward a variety of amino acids with coordinating side chain residues revealed rapid reactivity and selectivity for cysteine. To illustrate the potential utility of these compounds, their reactivity toward SARS-CoV-2-associated cysteine proteases is presented. Several SARS-CoV-2-associated proteins are cysteine proteases, including the 3-chymotrypsin-like protease (3CL^pro^, also termed the main protease M^pro^), papain-like protease (PL^pro^), human cathepsin B (CatB), and human cathepsin L (CatL).^23^ 3CL^pro^ and PL^pro^ are essential for proteolytic processing of the viral polyproteins. Inhibition of 3CL^pro^ or PL^pro^ can disrupt the viral life cycle, presenting a target for therapeutic intervention.^24−26^ The use of organic, covalent 3CL^pro^ and PL^pro^ inhibitors as antiviral agents has been reported.^27−29^ As alternative targets, the human enzymes CatB and CatL are responsible for cleavage of the SARS-CoV-2 spike protein and are necessary for entry of the virus into the host cell; therefore, inhibition of these enzymes can block viral internalization into host cells and hinder infectivity.^30,31^ The findings reported here show that Re^V^ complexes can rapidly react with all of these enzyme targets, forming well-characterized cysteine adducts as demonstrated by protein mass spectrometry and inductively coupled plasma mass spectrometry. This coordinate covalent modification leads to Re^V^ compounds that display apparent IC_50_ values as low as 9 nM toward SARS-CoV-2 cysteine proteases, further verifying the high reactivity of these compounds toward the tested enzymes. The tridentate S,S,S spectator ligand in these Re^V^ complexes was replaced with a S,N,S spectator ligand to provide a synthetic handle (i.e., carboxylic acid, boronic ester, alkyne, or bromide) that can facilitate incorporation of these reactive fragments into more elaborate and selective covalent inhibitors. Taken together, these findings suggest that Re^V^ complexes may serve as attractive, inorganic “metallofragment” alternatives for cysteine-targeting warheads.

## Results and Discussion

### Compound Synthesis and Reactivity

A total of eight Re^V^ compounds were prepared possessing a common S,S,S spectator ligand, but a variable labile ligand. The synthesis of complexes **1**,^32^**2**,^33^**4**,^34^**5**,^34^**7**,^19^ and **8**(19) has been previously reported; however, a different synthetic strategy was employed for their preparation in this study (see the Supporting Information for details). To the best of our knowledge, compounds **3** and **6** have not been previously reported (Figure 1c). The identity of the compounds was confirmed by NMR spectroscopy and mass spectrometry, and the purity of all compounds was verified by HPLC.

The aqueous solubility of the metal complexes is a crucial requirement for any biological application. Due to the poor water solubility of the compounds, stock solutions were prepared in dimethyl sulfoxide (DMSO). To assess the solubility of the metal complexes under physiological conditions, compounds **1**, **3**, and **5** were dissolved in DMSO and diluted with phosphate-buffered saline reaching 2% DMSO and a final compound concentration of 200 μM. No aggregation or particle formation was observed by dynamic light scattering, indicative of sufficient aqueous solubility of the metal complexes under these conditions. To be useful for potential therapeutic applications, the stability of aqua complex **3** was assessed in phosphate-buffered saline (PBS), as it represents the therapeutically active fragment (upon release of a labile ligand) for all of the complexes tested (**1**–**8**). Complex **3** was incubated in PBS at 37 °C for 48 h in the dark and analyzed by HPLC (Figure S1). No changes in the chromatograms were observed, suggesting that **3** is stable and does not degrade or further change under these conditions.

**Figure 2:**
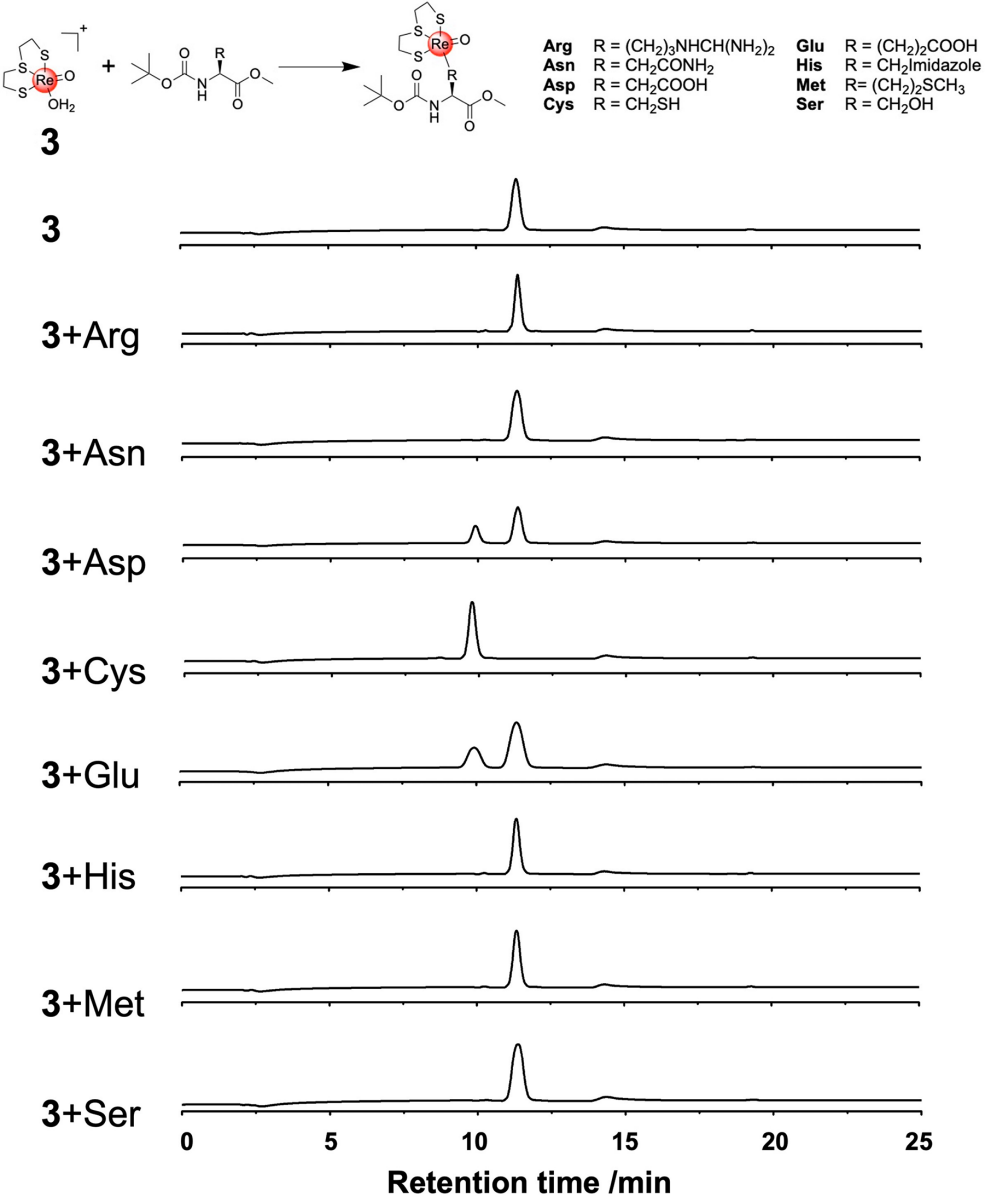
Top: Potential reactivity of Re^V^ complex **3** with various amino acids. Coordination via the amino acid residue (R group) occurs through the respective side chain heteroatoms. Bottom: HPLC traces upon incubation of **3** (0.1 mg/mL) with various amino acids in water at 37 °C for 24 h in the dark.

The reactivity of complex **3** with several amino acid derivatives was explored. Amino acid derivatives with cationic, anionic, polar, and sulfur-containing side chains were tested. Briefly, compound **3** (0.1 mg/mL) was incubated in equimolar amounts with the respective amino acid in water at 37 °C for 24 h in the dark. After incubation, the mixture was analyzed by HPLC to determine whether the metal complex formed adducts with the amino acids (Figure 2). While the complex did not show any reactivity toward the majority of the amino acids, **3** showed slow reactivity toward *tert*-butoxycarbonyl-l-aspartic acid methyl ester or *tert*-butoxycarbonyl-l-glutamic acid methyl ester but did not reach full conversion during the 24 h incubation period. Even upon prolonged incubation of *tert*-butoxycarbonyl-l-aspartic acid methyl ester with **3** for 72 h, full conversion was not achieved. By contrast, compound **3** produced a single product peak with full conversion upon incubation with *tert*-butoxycarbonyl-l-cysteine methyl ester. Using mass spectrometry, the identity of Re(2,2′-thiodiethanethiolate)(*tert*-butoxycarbonyl-l-cysteine methyl ester) ([M + H]^+^ calcd for C_13_H_25_NO_5_ReS_4_ 590.0, found 590.2) and Re(2,2′-thiodiethanethiolate)(*tert*-butoxycarbonyl-l-aspartic acid ester) ([M + H]^+^ calcd for C_14_H_25_NO_7_ReS_3_ 602.0, found 601.7) was verified. The reactivity of **3** was also tested with a shorter incubation time of 4 h toward *tert*-butoxycarbonyl-l-aspartic acid methyl ester and *tert*-butoxycarbonyl-l-cysteine methyl ester (Figure S2). Under these conditions, compound **3** did not show a significant amount of adduct formation with *tert*-butoxycarbonyl-l-aspartic acid methyl ester but did show complete conversion to an adduct with *tert*-butoxycarbonyl-l-cysteine methyl ester, indicative of its higher reactivity toward cysteine.

The ability of compounds **1**–**5** to react with cysteine residues was quantitatively assessed. The metal complexes (0.1 mg/mL) were incubated at 37 °C in the dark with equimolar amounts of *tert*-butoxycarbonyl-l-cysteine methyl ester in water, and the conversion was monitored over time by HPLC analysis. Aqua complex **3** showed the fastest reaction rate *(k* = (2.6 ± 1.0) × 10^–4^ M^–1^ s^–1^) among the Re^V^ complexes. Halogen-coordinated metal complexes **1** and **2** were found to display approximately half of the reaction rate of **3** (for **1**, *k* = (9.0 ± 1.8) × 10^–5^ M^–1^ s^–1^; for **2**, *k* = (5.2 ± 1.3) × 10^–5^ M^–1^ s^–1^). The reaction rates for **1**–**3** with cysteine were found to be quite comparable to those for typical organic warheads used to form covalent adducts with cysteine, such as acrylophenone (*k* = (9.3 ± 3.3) × 10^–4^ M^–1^ s^–1^) or phenylacrylamide (*k* = (1.1 ± 0.2) × 10^–4^ M^–1^ s^–1^).^35^ Thiolate-coordinated metal complexes **4** and **5** were found to react more than an order of magnitude slower with cysteine (for **4**, *k* = (6.2 ± 3.4) × 10^–6^ M^–1^ s^–1^; for **5**, *k* = (8.4 ± 2.6) × 10^–6^ M^–1^ s^–1^). Although the reaction was much slower, the thiolate-coordinating moiety of compounds **4** and **5** could be displaced upon incubation with this cysteine derivative, suggesting that the nature of the leaving ligand can be used to tune the rate of reactivity of the resulting Re^V^ complexes.

### Reactivity of Re^V^ Complexes toward SARS-CoV-2-Associated Cysteine Proteases

Complex **3** was incubated with either 3CL^pro^ or PL^pro^ for 2 h and analyzed by electrospray ionization mass spectrometry (ESI-MS,see the Supporting Information for details). CatB and CatL were not efficiently ionized under ESI-MS conditions and could not be evaluated by this technique. As a complementary experimental method, the binding of **3** was investigated by inductively coupled plasma mass spectrometry (ICP-MS). For ICP-MS analysis, complex **3** was incubated with the proteins of interest for either 2 or 6 h, followed by digestion with nitric acid and analysis of the metal ion content (Re and Zn) (see the Supporting Information for details). Unlike ESI-MS, ICP-MS proved to be a suitable analysis method for all of the proteins studied (3CL^pro^, PL^pro^, CatB, and CatL).

**Figure 3:**
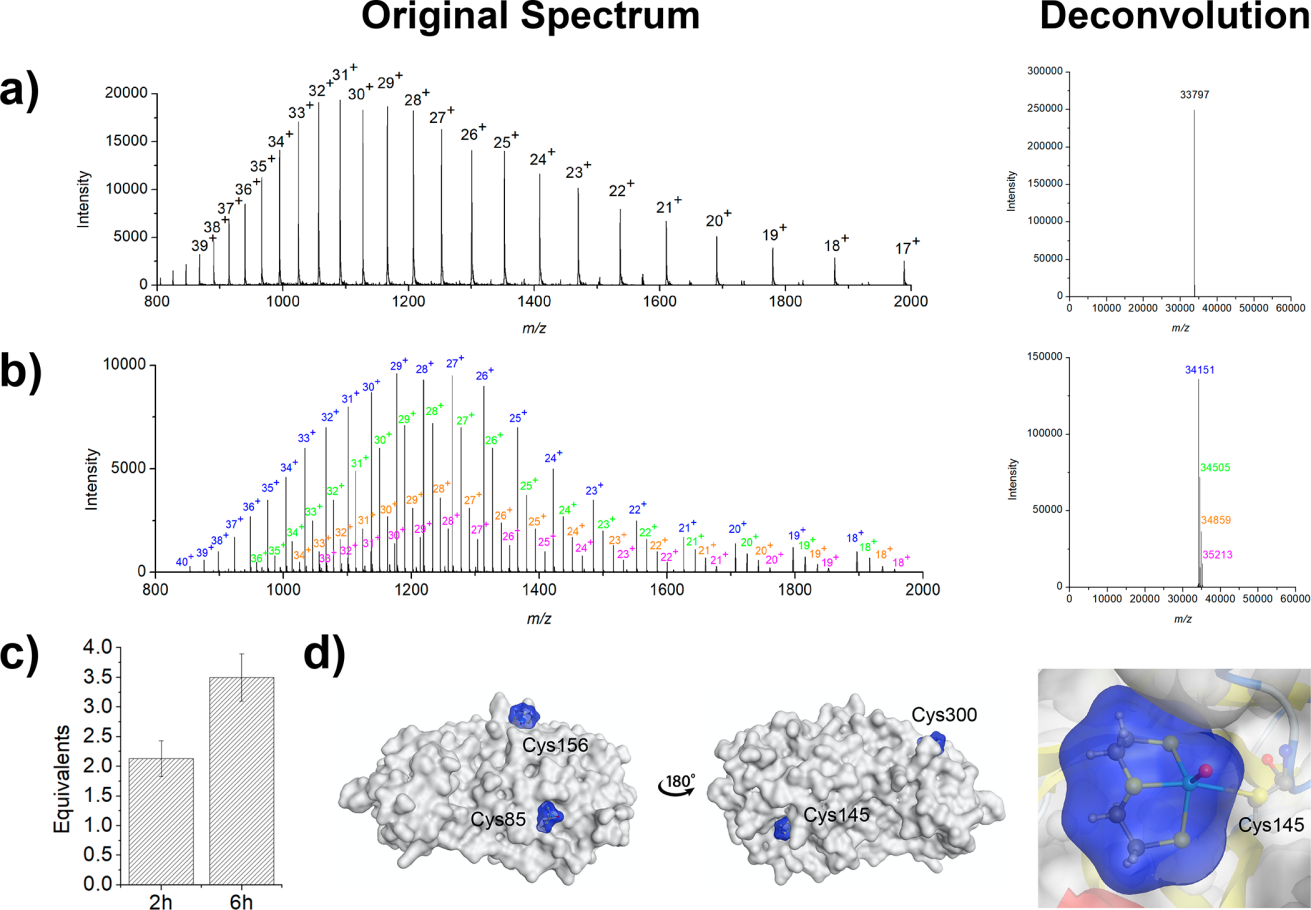
Coordinative covalent binding of metallofragment **3** to 3CL^pro^. Original and deconvoluted mass spectra of (a) 3CL^pro^ and (b) 3CL^pro^ upon incubation with **3**. The spectrum of 3CL^pro^ incubated with **3** shows a distribution of one (blue), two (green), three (orange), and four (pink) Re^V^ metallofragments bound to 3CL^pro^. (c) Determination of the Re content of 3CL^pro^ via ICP-MS analysis after co-incubation with the Re metallofragment for 2 or 6 h. (d) Predicted docking poses of the Re^V^ metallofragments with cysteine residues on 3CL^pro^ (PDB entry 6Y2F) visualized by protein surface representations. The zoomed image is of the docking pose with the Cys145 residue of 3CL^pro^.

The mass spectrum of 3CL^pro^ alone shows a distribution of various charge states that upon deconvolution give an *m*/*z* of 33 797 Da (Figure 3a). Upon incubation with aqua complex **3**, several protein–fragment adducts are formed with 3CL^pro^. The deconvoluted spectrum shows a distribution of one (blue), two (green), three (orange), and four (pink) Re^V^ metallofragments (the mass of a single metallofragment is 354 Da) bound to 3CL^pro^ (Figure 3b). 3CL^pro^ has 12 cysteine residues (Cys16, Cys22, Cys38, Cys44, Cys85, Cys117, Cys128, Cys145, Cys156, Cys160, Cys265, and Cys300), but only five of these are surface accessible (Cys44, Cys85, Cys145, Cys156, and Cys300). Notably, Cys44 and Cys145 are found very close together in the active site, likely allowing for binding of the metal complex to only one of these residues. Quantification of the number of Re^V^ complexes coordinated to 3CL^pro^ was also evaluated by ICP-MS. After incubation with **3** for 2 h (the same incubation time used for the ESI-MS experiments), an average of 2.1 ± 0.3 equiv of Re was found to be bound to 3CL^pro^. Notably, this value agrees with the MS analysis upon weighting the respective peaks toward the amount of metal complex bound (∼1.8 equiv of Re). Interestingly, upon incubation of the protein with **3** for 6 h, an average of 3.5 ± 0.4 equiv of Re was found coordinated to 3CL^pro^, corresponding to an increase in the average number of metal adducts coordinated to the protein (Figure 3c).

For experimental verification that the metallofragment can target Cys residues in the active site (Cys44 and Cys145), 3CL^pro^ was first incubated with the well-known covalent inhibitor GC376, which has been crystallographically characterized to bind to Cys145.^27^ As expected, upon incubation of 3CL^pro^ with GC376 spectral deconvolution gave a peak with an *m*/*z* of 34 201 Da, corresponding to a mass difference of 404 Da, and indicates that a single GC376 molecule is attached to 3CL^pro^ (Figure S3). Following this, the generated GC376–protein adduct was incubated with Re^V^ complex **3** and the distribution of protein–fragment adducts was obtained. The spectrum shows a distribution of the coordinative covalent binding of one (blue), two (green), and three (orange) metallofragments bound to the GC376–3CL^pro^ adduct (Figure S4). Preincubation with GC376 results in 1 equiv fewer of the Re^V^ complex being bound, indicating that the metal complex binds to a Cys residue in the active site (Cys44 or Cys145) of 3CL^pro^. To identify the sites of adduct formation, 3CL^pro^ was incubated with compound **3** for 4 h. The modified protein was isolated by sodium dodecyl sulfate–polyacrylamide gel electrophoresis (SDS–PAGE); the isolated protein was digested with trypsin, and the binding sites were identified by analysis of the generated peptide fragments by ultra-high-pressure liquid chromatography (UPLC) coupled with tandem mass spectroscopy (LC-MS/MS), as previously reported for organic covalent binders.^36^ In agreement with the ESI-MS analysis, four binding sites (Cys44, Cys85, Cys156, and Cys300) for **3** were identified (Table 1). Amino acids Cys44, Cys85, Cys156, and Cys300 are all surface accessible residues, with Cys44 found in the active site. It is important to note that Cys44 is not the catalytically essential Cys residue for 3CL^pro^, which is Cys145, but was an anticipated site of adduct formation.

**Table 1:** Modified Cys Residues upon Incubation of 3CL^pro^ with **3** Determined by Protein Digestion Analysis^a^

peptide	site of labeling	function
H.VIC[**3**]TSEDMLNPNYEDLLIR.K	Cys44	active site Cys
R.VIGHSMQNC[**3**]VLK.L	Cys85	surface accessible Cys
D.YDC[**3**]VSFCYMHHMELPTGVHAGTDLEGNFYGPFVDR.Q	Cys156	surface accessible Cys
R.TILGSALLEDEFTPFDVVRQC[**3**]SGVTF.Q	Cys300	surface accessible Cys

a Re(V)-modified residues are indicated by the C[**3**] designation. Periods at the start and end of each sequence indicate peptide cleavage sites. Experimental details can be found in the Experimental Section.

The ESI-MS and ICP-MS data support the binding of ≤4 equiv of **3** to 3CL^pro^, which represents all surface accessible cysteine residues (vide supra). To gain some molecular insight into the nature of these adducts, the binding poses of the metal complexes bound to 3CL^pro^ were computationally modeled. The geometry of the metallofragment complex was optimized using density functional theory (DFT) calculations (see the Supporting Information for details). The labile water ligand was removed, and the resulting structure was fixed as a rigid body and the metal complex was covalently docked toward Cys residues found in 3CL^pro^ (PDB entry 6Y2F). As expected from the experimental data, the metallofragment was found to form coordinate covalent adducts with Cys85, Cys156, and Cys300 (Figure 3d). The docking experiment indicated that the Re^V^ metallofragment could coordinate to Cys145; however, the experimental determination of the binding sites by protein digestion analysis revealed binding to the Cys44 residue (Table 1). To examine this further, the binding poses of **3** with Cys44 and Cys145 were computationally modeled by Cys–metal center formation with flexible amino acid side chains (Figure S5). On the basis of the steric demand of the complex, only one metal adduct can fit into the active site; the formation of both adducts would be sterically occluded. A comparison of the predicted energetic levels indicated that the binding of **3** to Cys145 is energetically favored by ∼0.9 kcal/mol. As the energetic difference between these binding poses is small, it is reasonable to conclude that both adducts could be accessible,^37^ although only the Cys44 adduct is experimentally observed as described above (Table 1).

On the basis of the large number of Re^V^ complexes coordinated to 3CL^pro^, this protein was used to study the reversibility of the cysteine-targeting warhead. To challenge the coordinate covalent interaction, 3CL^pro^ (7.4 μM) was incubated with **3** (50 μM) for 6 h and the metal–3CL^pro^ adduct was isolated using a molecular weight cutoff filter (10 kDa MWCO). The isolated protein was washed to remove excess unbound metal complexes and then exposed for 2 h to an excess of *tert*-butoxycarbonyl-l-cysteine methyl ester (0.25 mM)^38^ or glutathione (2 mM),^39^ which corresponds to the level of each biologically relevant thiol in mammalian cells. After incubation, the Re^V^-modified 3CL^pro^ was washed and the metal content of the enzyme was determined by ICP-MS. An average of 3.1 ± 0.4 equiv of Re was found to be coordinated to 3CL^pro^. This value is in the same range as that of the enzyme that was not challenged (3.5 ± 0.4 equiv of Re) with biological thiols, suggesting that once formed, these Re^V^–Cys adducts are stable.

**Figure 4:**
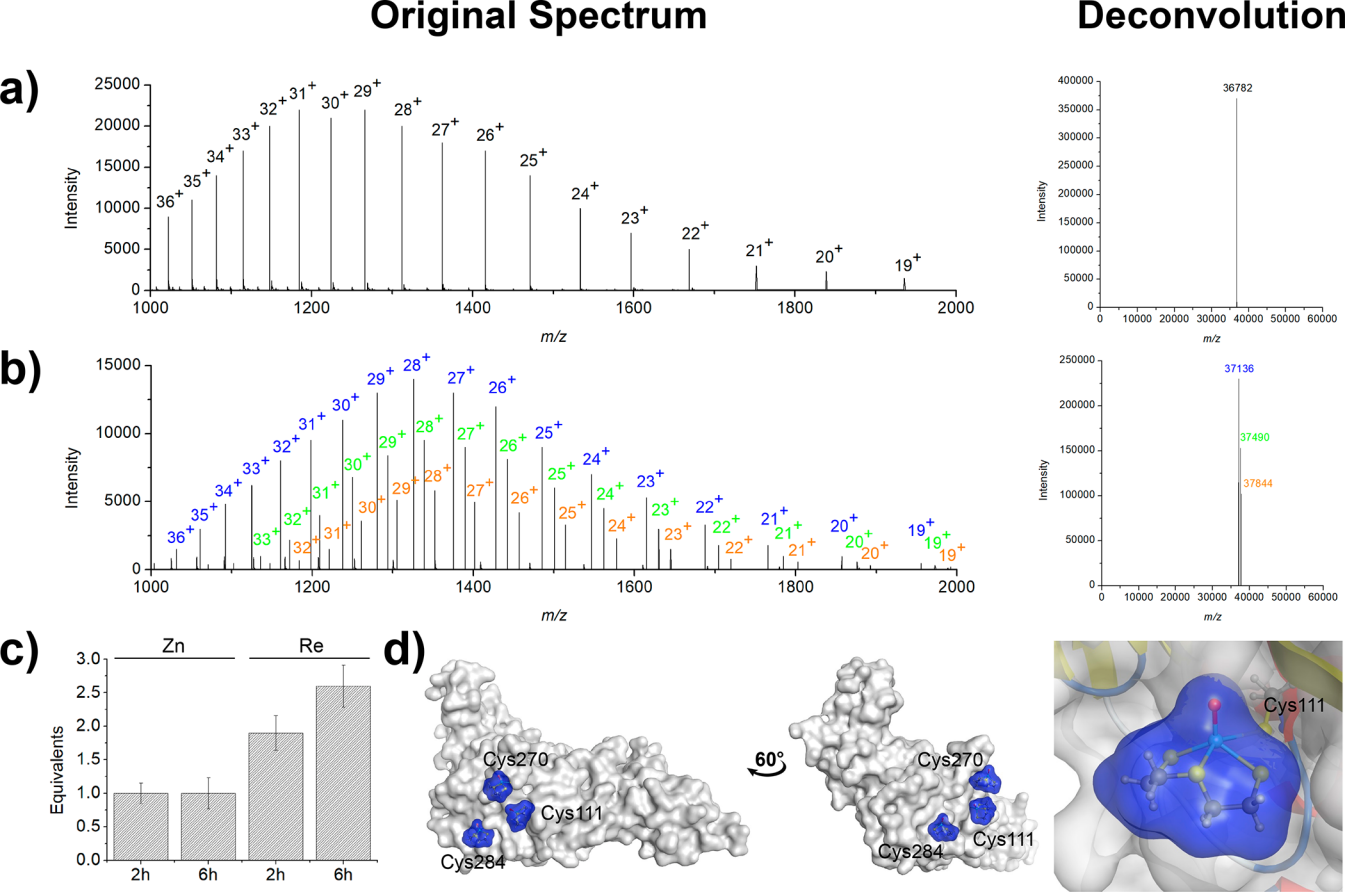
Coordinative covalent binding of metallofragment **3** to PL^pro^. Original and deconvoluted mass spectra of (a) PL^pro^ and (b) PL^pro^ upon incubation with **3**. The spectrum of PL^pro^ incubated with **3** shows a distribution of the coordinative covalent binding of one (blue), two (green), and three (orange) metallofragments bound to PL^pro^. (c) Determination of the Re and Zn content of PL^pro^ via ICP-MS analysis after co-incubation with the Re metallofragment for 2 or 6 h. (d) Predicted docking poses of the Re^V^ metallofragments with cysteine residues on PL^pro^ (PDB entry 7NFV) visualized by protein surface presentation. The zoomed image is of the docking pose with the Cys111 residue of PL^pro^.

The ability of metallofragment **3** to bind PL^pro^ was similarly investigated. The mass spectrum of the native PL^pro^ enzyme is characterized by a distribution of masses that upon deconvolution correspond to an *m*/*z* of 36 782 Da (Figure 4a). Upon incubation with **3**, a mixture of protein–inhibitor adducts was obtained with one (blue), two (green), and three (orange) Re^V^ complexes bound to PL^pro^ (Figure 4b). An analysis of PL^pro^ shows that the enzyme possesses 11 cysteine residues (Cys111, Cys148, Cys155, Cys181, Cys189, Cys192, Cys224, Cys226, Cys260, Cys270, and Cys284). Among these, Cys189, Cys192, Cys224, and Cys226 are ligands for a structural Zn(II) site and only residues Cys111, Cys270, and Cys284 are solvent accessible for metallofragment binding, which is consistent with the ESI-MS findings.

The metal content of PL^pro^ upon incubation of **3** was also evaluated by ICP-MS analysis. As noted above, PL^pro^ is a metalloenzyme with a structural Zn(II) ion bound by cysteine residues (Cys189, Cys192, Cys224, and Cys226). It has been suggested that some Au(I)/(III) complexes can displace the Zn(II) ion and coordinate to these Cys residues of PL^pro^, thereby inhibiting the enzyme.^40,41^ To investigate the potential of the Re^V^ complexes to interact in a similar manner, the Re and Zn content of the enzyme was determined by ICP-MS in an identical procedure as described above for 3CL^pro^. Upon incubation with **3**, no changes in the Zn content of PL^pro^ were observed, suggesting that the Zn(II) ion is not released upon modification by Re^V^ metallofragments (Figure 4c). In agreement with the ESI-MS data, after incubation of **3** with PL^pro^ for 2 h, an average of 1.9 ± 0.3 equiv of Re was found to be bound to the enzyme by ICP-MS. Extending the incubation time to 6 h results in an average of 2.6 ± 0.3 equiv of the metallofragment coordinated to the protein (Figure 4c). Computational docking was performed to determine the possible sites of binding of **3** with PL^pro^. In agreement with the experimental data, the metallofragment was found to form adducts with Cys111, Cys270, and Cys284 (Figure 4d). As compound **3** is predicted to bind to the catalytically active Cys111 residue in the catalytic triad (Cys111-His272-Asp286), this metallofragment is expected to inhibit the activity of PL^pro^.

The human enzyme CatB did not efficiently ionize under the ESI-MS conditions used, and as such, it was not possible to investigate the binding of **3** by ESI-MS. However, binding of **3** to CatB was studied by ICP-MS. Upon incubation of **3** for 2 or 6 h, an average of 1.3 ± 0.2 or 1.8 ± 0.3 equiv of Re was found bound to CatB (Figure 4a). CatB possesses 14 cysteine residues (Cys14, Cys26, Cys29, Cys43, Cys62, Cys63, Cys67, Cys71, Cys100, Cys108, Cys119, Cys128, Cys132, and Cys240). Despite the large number of cysteine residues in CatB, the majority of these form disulfide bonds or are in the interior of the protein. Only residues Cys29 and Cys240 can be accessed from the surface and could present sites of adduct formation. This is consistent with the ICP-MS data, and docking experiments were used to show the adducts of **3** bound to these two residues (Figure 4b). As the compound was predicted to interact with the catalytic Cys29 residue, it is expected that the metal complex should be able to inhibit the activity of CatB.

In a similar manner, the binding of **3** to human CatL was studied by ICP-MS. The analysis of the metal content revealed that upon incubation of **3** with CatL for 2 and 6 h an average of 0.6 ± 0.2 and 1.0 ± 0.1 equiv of Re were coordinated to the protein (Figure 4c). CatL possesses seven cysteine residues (Cys22, Cys25, Cys56, Cys65, Cys98, Cys156, and Cys209), but the structure shows that only Cys25 is located on the surface. Computational docking experiments confirm that a coordinate covalent interaction with **3** was formed with only Cys25 (Figure 4d). As Cys25 is the key catalytic residue, it is predicted that **3** should inhibit CatL.

### Inhibition of SARS-CoV-2-Associated Cysteine Proteases

Having determined that compound **3** could form coordinate covalent adducts with SARS-CoV-2-associated cysteine proteases, we investigated the ability of these Re^V^ metallofragment warheads to inhibit 3CL^pro^, PL^pro^, CatB, and CatL (Table 2 and Figure S6). Complexes **1**–**8** were incubated with each enzyme, and activity was monitored by the conversion of a nonfluorescent substrate to a fluorescent product (see the Supporting Information for details). Re^V^ complexes **1**–**3** were found to strongly inhibit 3CL^pro^, CatB, and CatL with apparent IC_50_ values in the low nanomolar range (IC_50_ = 9–51 nM) and in the micromolar range for PL^pro^ (IC_50_ = 7.6–22.4 μM). The Re^V^ complexes with thiolate moieties as capping groups (**4**–**8**) showed a significantly weaker inhibition, likely because of the slower reaction kinetics (vide supra), which highlights the role of the leaving group. As a general trend, it was observed that the inhibitory activity decreased with an increase in the steric demand of the labile ligand. This may be due to the inability of the bulkier complexes to access and undergo nucleophilic attack by the Cys residues. For example, the sterically demanding 4-phenylbenzene-1-thiolate-functionalized complex **6** was found to be inactive toward all of the cysteine proteases. Notably, all of the metallofragments showed significantly weaker inhibition of PL^pro^ compared to that of the other cysteine proteases tested. Overall, **3** displayed the strongest inhibitory effect (IC_50,3CL^pro^_ = 18 ± 6 nM, IC_50,CatB_ = 9 ± 3 nM, and IC_50,CatL_ = 19 ± 5 nM). These results highlight the ability of **3** to inhibit the activity of these enzymes.

**Table 2:** Half-Maximal Inhibitory Concentrations (IC_50_) of Metal Complexes **1**–**8** with Respect to the SARS-CoV-2-Associated Cysteine Proteases 3-Chymotrypsin-like Protease (3CL^pro^), Papain-like Protease (PL^pro^), Cathepsin B (CatB), and Cathepsin L (CatL)^a^

	3CL^pro^ (μM)	PL^pro^ (μM)	CatB (μM)	CatL (μM)
**1**	0.037 ± 0.008	19.6 ± 3.3	0.016 ± 0.003	0.026 ± 0.006
**2**	0.046 ± 0.009	22.4 ± 4.7	0.018 ± 0.005	0.051 ± 0.007
**3**	0.018 ± 0.006	7.6 ± 2.8	0.009 ± 0.003	0.019 ± 0.005
**4**	0.75 ± 0.17	>100	9.3 ± 2.7	0.153 ± 0.007
**5**	1.43 ± 0.20	>100	>100	0.186 ± 0.009
**6**	>100	>100	>100	>100
**7**	0.84 ± 0.26	>100	>100	0.194 ± 0.007
**8**	59.4 ± 6.3	>100	>100	>100

a Values and standard deviations are derived from three independent experiments. Experimental details for all enzymatic assays can be found in the Experimental Section.

### Selectivity against Off-Target Human Proteases

To evaluate the selectivity of these warheads for nontargets, the activity of compounds **3** (the most active compound) and **5** (which showed strong inhibition and selectivity for 3CL^pro^ and CatL) was tested against human serine protease dipeptidyl peptidase-4 (DPP4), aspartate protease β-secretase 1 (BACE1), and the serine protease furin. While DPP4, BACE1, and Furin do not have a catalytically active cysteine residue, these enzymes have surface accessible cysteine residues (Table S1) where the metallofragments could potentially bind. Recent studies have indicated the involvement of furin in the cellular uptake of SARS-CoV-2.^42^ At a concentration of 50 μM, neither metallofragment (**3** or **5**) showed inhibition of DPP4, BACE1, or Furin, suggesting that the inhibition observed by these fragments against cysteine-dependent proteases is the result, at least in part, of complexation with active site Cys residues. In addition, these data suggest that selectivity can be obtained over other human proteases that are not dependent on Cys (Figure S7).

### Derivatization of the Spectator Ligand Scaffold

**Scheme 1:**
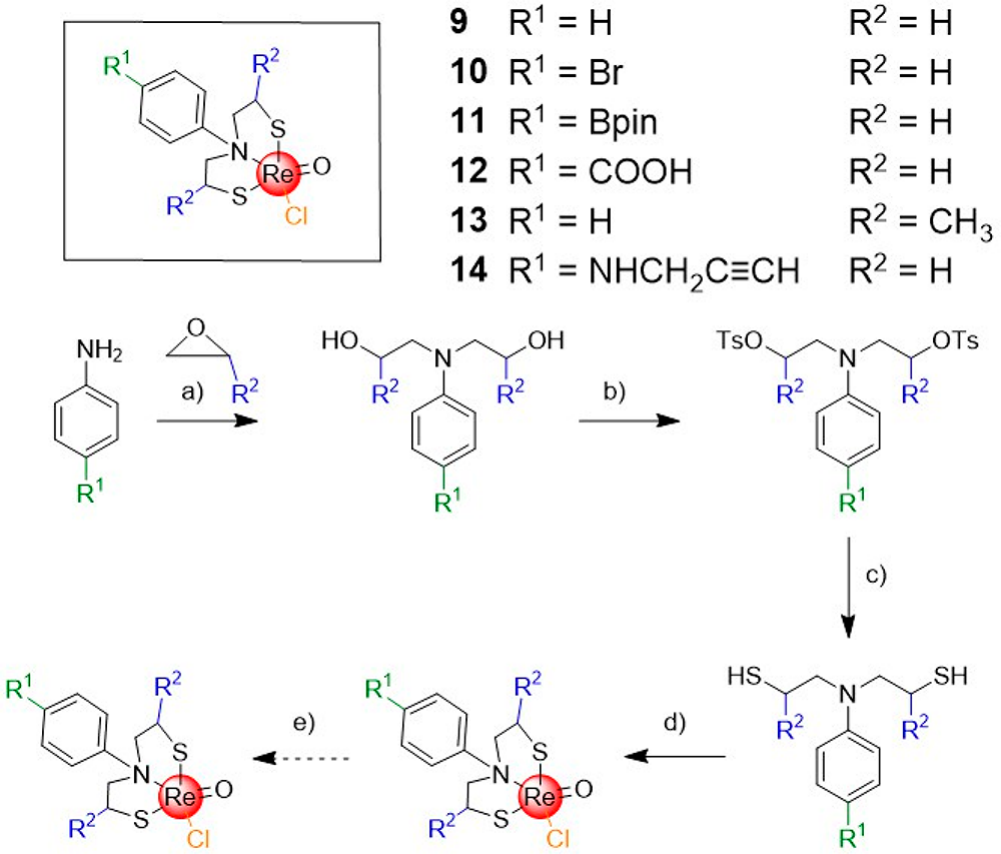
Synthetic Route for the Derivatization of Metal Complexes Based on Re[*N*,*N*-bis(2-thioethyl)aniline](chloride) Conditions: (a) water, catalytic amounts of propionic acid, 0 °C, room temperature, overnight; (b) dry pyridine/dichloromethane, 4-methylbenzenesulfonyl chloride, room temperature, overnight; (c) thiourea, ethanol, reflux, 3 h, sodium bicarbonate, reflux, 4 h, or thiolactic acid, triethylamine, dry tetrahydrofuran, room temperature, overnight, hydrogen chloride solution in water, room temperature, overnight; (d) tetrabutylammonium tetrachlorooxorhenate(V), methanol, chloroform, room temperature, overnight; (e) thiol, darkness, room temperature, 2 h.

To use these Re^V^ metallofragments as warheads in covalent enzyme inhibitors, their elaboration into more sophisticated molecular structures is necessary. While the chemical literature reports on synthetic routes for the preparation of derivatives of the S,S,S 2,2′-thiodiethanethiol scaffold,^43−45^ these synthetic procedures are challenging and limited with respect to accessible functional groups. To overcome this drawback, the center thiol atom of the ligand was replaced with an amine nitrogen atom (Scheme 1) as a synthetically more tractable scaffold. The S,N,S tridentate ligand is prepared by a nucleophilic attack of the nitrogen atom of an aniline derivative with an epoxide. The hydroxy groups obtained during ring opening of the epoxide are then functionalized with toluenesulfonyl groups. In the presence of thiourea or thiolactic acid, thiol groups can be installed with the release of toluenesulfonic acid. Finally, upon addition of the tetrachlorooxorhenate(V) precursor, the desired Re^V^ complexes can be generated (see the Supporting Information for details). As examples for the versatile derivatization of the core scaffold, several derivatives with functionalized anilines (**9**–**12**, R^1^) or a functionalized 2-thioethyl moiety (**13**, R^2^) were prepared. Among these, several common synthetic “handles” [Br, **10**; boron(pinacolato) (Bpin), **11**; COOH, **12**] were installed on the aniline moiety that could be used for derivatization or conjugation to obtain compounds with potentially improved target selectivity.

To ensure that the derivatization of the central thiol with an amine did not unfavorably alter the bioactive properties of these compounds, the formation of an adduct of **9** with *tert*-butoxycarbonyl-l-cysteine methyl ester was monitored in a time-dependent manner by HPLC and the reaction rate determined. Complex **9** formed an adduct with cysteine at a rate of (4.2 ± 1.3) × 10^–5^ s^–1^, which is in the same range as for the analogous compound **1** (*k* = (6.1 ± 1.2) × 10^–5^ s^–1^), suggesting that the derivatization of the central thiol group does not impede the ability of the Re^V^ center to bind cysteine residues. To further assess the biochemical properties of this compound, the ability of **9** to inhibit 3CL^pro^ and CatB was measured. Complex **9** was found to exhibit strong inhibition of these enzymes (IC_50,3CL^pro^_ = 68 ± 9 nM, and IC_50,CatB_ = 44 ± 6 nM). To verify the coordinate covalent adduct, 3CL^pro^ was incubated with **9** and adduct formation was probed by ESI-MS (vide supra). Interestingly, upon deconvolution a single peak with a *m*/*z* of 34 210 Da, corresponding to a mass difference of 413 Da, was observed, indicating that a single Re^V^ metallofragment was attached to the protein (Figure S8). Because compound **9** could effectively inhibit 3CL^pro^, which suggested that the metal complex is coordinated to the active site, 3CL^pro^ was first incubated with GC376 (Figure S3) and afterward incubated with **9**. No additional adduct from **9** was observed (Figure S9), suggesting that **9** targets the active site residues (either Cys44 or Cys145, see above). Overall, these findings show the potential of Re^V^ complexes with a modifiable spectator ligand to function as warheads toward cysteine-dependent enzymes.

To evaluate whether these metal complexes can penetrate human cells, a parallel artificial membrane permeability assay was performed using a previously reported protocol (see the Supporting Information for details).^46^ Metal complexes **1** (0.038 ± 0.005 μm/s), **3** (0.044 ± 0.006 μm/s), and **9** (0.036 ± 0.006 μm/s) were found to exhibit high permeability rates, based on comparisons to control compounds with established rates of permeability.

Previous studies of organic warheads have demonstrated that a warhead can show a unique reactivity profile against a mixture of proteins. Literature studies have focused on iodoacetamide, maleimide, or acrylate-based warheads.^47^ For a preliminary evaluation of the complexes reported here, the labeling of specific proteins within the proteosome was studied by fluorescently labeled SDS–PAGE. A reported protocol was employed, and the known organic warhead oct-1-en-7-yn-3-one was used as a reference compound.^48^ Before the labeling of the proteome was attempted, the conditions of the labeling were optimized by incubating free cysteine with the warhead oct-1-en-7-yn-3-one or compound **14**, a derivative that is functionalized with a pendant alkyne group (Scheme 1). Incubating either compound with an azide-functionalized rhodamine dye, copper(II) sulfate, and sodium ascorbate results in a click reaction between the dye and warhead, as evidenced by mass spectrometry (data not shown).

A mouse proteome was obtained using heart, lung, kidney, intestine, and liver tissue from 4-week-old C57Bl6/N female mice. The proteome was incubated with the warhead oct-1-en-7-yn-3-one or **14** for 2 h. Next, the proteome mixture was further incubated with the rhodamine dye, copper(II) sulfate, and sodium ascorbate for an additional 2 h, resulting in the labeling of several proteins as visualized by fluorescence imaging of the resulting SDS–PAGE gel (Figure S10a). Control experiments that involved incubation of the proteosome with dimethyl sulfoxide, the warhead alone, or the fluorescent dye alone showed no protein labeling. To verify the composition of the proteosome, gels were also stained with Coomassie to visualize all proteins (Figure S10b). The comparison between the warheads showed that oct-1-en-7-yn-3-one labeled more proteins than **14**, consistent with the very simple structure and expected low specificity of oct-1-en-7-yn-3-one. In addition, labeling of the proteome with **14** was studied in a concentration-dependent manner. The resulting SDS–PAGE gel shows distinctive bands indicative of a preliminary selectivity for some proteins (Figure S11). Future studies could elaborate on the identification of these specific proteins.

## Conclusion

In summary, Re^V^ metallofragments with a stable tridentate spectator ligand and a labile, monodentate ligand were evaluated as Cys-targeting warheads for coordinate covalent inhibition. These metal complexes were found to rapidly bind to Cys residues with a binding rate that is comparable to those of organic warheads used for targeting of cysteine in covalent inhibitors. The binding of a Re^V^ metallofragment with various SARS-CoV-2-associated cysteine proteases was verified by ESI-MS and ICP-MS experiments, as well as computational modeling. Importantly, adduct formation was localized to surface Cys residues, including active site Cys residues. Consistent with active site adduct formation, the metallofragments were found to inhibit SARS-CoV-2-associated cysteine proteases. To enable the utility of this class of metallofragment warheads, the central thiol of the tridentate spectator ligand was replaced with an amine and the derivatization of the core scaffold with different synthetic handles was demonstrated. Treatment of a mouse proteosome also provided encouraging results for the use of these complexes as coordinate covalent warheads. Despite these promising preliminary findings, the ability of these metal complexes to inhibit the SARS-CoV-2 virus has not been explored. It is expected that the compounds need to be further elaborated into a more druglike compound to be suitable for potential use as an antiviral agent. Collectively, these data indicate that Re^V^ metallofragments can function as a novel class of inorganic Cys-targeting warheads for SARS-CoV-2 cysteine proteases, as well as other Cys-dependent enzymes.

## Experimental Section

### General Synthetic Experimental Details

All reagents and solvents were obtained from commercial sources and used without further purification. Solvents were dried over molecular sieves if necessary. NMR spectra were recorded with apparatus from the nuclear magnetic resonance facility located in the Department of Chemistry and Biochemistry at the University of California, San Diego. ^1^H and ^13^C NMR spectra were recorded on a 400 MHz NMR spectrometer. The spectra were analyzed by chemical shifts (δ) in parts per million referenced to tetramethylsilane (δ 0.00) using the residual proton solvent peaks as internal standards and coupling constants (*J*) in hertz. The multiplicity of the peaks is abbreviated as follows: br, broad; s, singlet; d, doublet; t, triplet; m, multiplet. Mass spectra were recorded at the molecular mass spectrometry facility located in the Department of Chemistry and Biochemistry at the University of California, San Diego. For analytical HPLC, the following system was used: Agilent 1200 series degasser and pump system with an Agilent Ecplise XDB-C18 (5 μm, 150 mm × 4.6 mm) column. The solvents (HPLC grade) were Millipore water (solvent A) and acetonitrile (solvent B). The solvent gradient was as follows: 0–3 min, isocratic 95% A (5% B); 3–17 min, linear gradient from 95% A (5% B) to 0% A (100% B); 17–25 min, isocratic 0% A (100% B). All metal complexes were found to be at least 95% pure as confirmed by HPLC and ^1^H NMR analysis. Infrared spectra were recorded on a Bruker Alpha II FTIR-ATR spectrometer. The intensity of the peaks is abbreviated as follows: br, broad; s, strong; m, medium; w, weak.

### Synthetic Procedure and Compound Characterization

#### Tetrabutylammonium Tetrachlorooxorhenate(V)

The compound was prepared using a reported protocol^49^ with minor modifications. Ammonium perrhenate(VII) (1.00 g, 3.7 mmol) was suspended in ethanol (20 mL), and tetrabutylammonium bromide added (2.38 g, 7.4 mmol). A solution of hydrogen chloride in diethyl ether (2 M, 10 mL) was slowly added, and the mixture was stirred at room temperature for 3 h. After this time, the solvent was removed and the crude mixture recrystallized from ethanol inside a freezer. The precipitate was collected by filtration and washed with diethyl ether (10 mL). Yield: 1.82 g (3.1 mmol, 84%). FT-IR: 2956 (brm), 2870 (brm), 1480 (m), 1382 (w), 1030 (brw), 992 (brw), 927 (s), 858 (s), 737 (m) cm^–1^.

#### ReO(2,2′-thiodiethanethiol)(chloride) (**1**)

The compound was prepared using a reported protocol^32^ with minor modifications. Tetrabutylammonium tetrachlorooxorhenate(V) (200 mg, 0.34 mmol) was dissolved in dry methanol (10 mL) under a nitrogen atmosphere. A solution of 2,2′-thiodiethanethiol (53 mg, 0.34 mmol) in dry chloroform (3 mL) was added dropwise. Immediate precipitation was observed. The mixture was stirred at room temperature for an additional 2 h, and after this time, the precipitate was isolated via filtration. The solid was washed with methanol (3 × 5 mL), dichloromethane (2 × 5 mL), and diethyl ether (5 mL). The compound was recrystallized from ethanol/dichloromethane (1:2) inside a freezer. The precipitate was collected by filtration and washed with diethyl ether (10 mL). Yield: 105 mg (0.27 mmol, 79%). FT-IR: 2966 (w), 2919 (w), 1415 (m), 1266 (m), 965 (s), 845 (m) cm^–1^. ^1^H NMR (400 MHz, CD_3_CN): δ 4.22–4.15 (m, 4H), 3.24 (ddd, *J* = 14.0, 13.2, 4.4 Hz, 2H), 2.57 (ddd, *J* = 14.3, 10.8, 5.1 Hz, 2H). ^13^C{^1^H} NMR (100 MHz, CD_3_CN): δ 51.6, 45.1. HR-MS (*m*/*z*): [M + H]^+^ calcd for C_4_H_9_ClOReS_3_, 390.9054; found, 390.9052. RP-HPLC: *t*_R_ = 16.2 min.

#### ReO(2,2′-thiodiethanethiol)(bromide) (**2**)

This compound has been previously reported,^33^ but a different synthetic procedure was used here. Re(2,2′-thiodiethanethiol)(chloride) (100 mg, 0.26 mmol) was dissolved in dry acetone (50 mL), and an excess of potassium bromide (309 mg, 2.6 mmol) added. The mixture was heated at reflux for 4 h in the dark. After this time, the solvent was removed under reduced pressure and the crude product purified by reverse phase column chromatography with a linear gradient (0%/100% to 100%/0% methanol/water). The fractions containing the product were combined, and the compound was dried under vacuum. Yield: 104 mg (0.24 mmol, 92%). FT-IR: 2963 (w), 2912 (w), 1416 (m), 1269 (m), 1237 (w), 965 (s), 847 (m) cm^–1^. ^1^H NMR (400 MHz, CD_3_CN): δ 4.20–4.12 (m, 4H), 3.21 (ddd, *J* = 13.8, 13.4, 4.4 Hz, 2H), 2.55 (ddd, *J* = 14.2, 10.8, 5.1 Hz, 2H). ^13^C{^1^H} NMR (100 MHz, CD_3_CN): δ 51.6, 45.0. HR-MS (*m*/*z*): [M + H]^+^ calcd for C_4_H_9_BrOReS_3_, 434.8549; found, 434.8551. RP-HPLC: *t*_R_ = 18.7 min.

#### ReO(2,2′-thiodiethanethiol)(water) Trifluoromethanesulfonate (**3**)

Re(2,2′-thiodiethanethiol)(chloride) (100 mg, 0.26 mmol) was dissolved in dry acetone (50 mL), and silver(I) trifluoromethanesulfonate (67 mg, 0.26 mmol) added. The reaction mixture was heated to reflux for 4 h in the dark. After this time, the precipitated silver chloride was removed by filtration. The solution was concentrated by evaporation upon reduced pressure. Millipore water (10 mL) was added, and the desired product precipitated. The solid was collected by filtration and washed with diethyl ether (10 mL). Yield: 99 mg (0.19 mmol, 73%). ^1^H NMR [400 MHz, (CD_3_)_2_CO]: δ 4.41 (dd, *J* = 13.7, 13.6, 4.4 Hz, 2H), 4.23 (dd, *J* = 13.8, 13.5, 5.0 Hz, 2H), 3.27 (ddd, *J* = 13.6, 13.5, 4.4 Hz, 2H), 2.55 (ddd, *J* = 13.8, 13.6, 5.0 Hz, 2H). ^13^C{^1^H} NMR (100 MHz, CD_3_CN): δ 51.5, 44.9. HR-MS (*m*/*z*): [M – triflate – H_2_O]^+^ calcd for C_4_H_8_OReS_3_, 354.9289; found, 354.9291. RP-HPLC: *t*_R_ = 11.4 min.

#### ReO(2,2′-thiodiethanethiol)(1-propanethiol) (**4**)

This compound has been previously reported,^34^ but a different synthetic procedure was used here. Re(2,2′-thiodiethanethiol)(water) trifluoromethanesulfonate (68 mg, 0.13 mmol) was dissolved in dry acetonitrile (25 mL), and 1-propanethiol (14 μL, 0.15 mmol) added. The reaction mixture was stirred at room temperature for 2 h in the dark. After this time, the solvent was removed under reduced pressure and the crude product purified by reverse phase column chromatography with a linear gradient (25%/75% to 100%/0% methanol/water). The fractions containing the product were combined, and the compound was dried under vacuum. Yield: 29 mg (0.07 mmol, 52%). ^1^H NMR [400 MHz, (CD_3_)_2_CO]: δ 4.40 (ddd, *J* = 13.7, 13.1, 2.8 Hz, 2H), 3.98 (t, *J* = 7.3 Hz, 2H), 3.71 (dd, *J* = 13.6, 13.2 Hz, 2H), 3.27 (ddd, *J* = 13.5, 13.1, 2.9 Hz, 2H), 2.58 (dd, *J* = 13.4, 13.1 Hz, 2H), 1.69–1.65 (m, 2H), 1.05 (t, *J* = 7.4 Hz, 3H). ^13^C{^1^H} NMR [100 MHz, (CD_3_)_2_CO]: δ 50.7, 44.0, 27.1, 25.9, 12.3. HR-MS (*m*/*z*): [M + H]^+^ calcd for C_7_H_16_OReS_4_, 430.9636; found, 430.9637. RP-HPLC: *t*_R_ = 13.6 min.

#### ReO(2,2′-thiodiethanethiol)(benzenethiol) (**5**)

This compound has been previously reported,^34^ but a different synthetic procedure was used here. Re(2,2′-thiodiethanethiol)(water) trifluoromethanesulfonate (68 mg, 0.13 mmol) was dissolved in dry acetonitrile (25 mL), and benzenethiol (15 μL, 0.15 mmol) added. The reaction mixture was stirred at room temperature for 2 h in the dark. After this time, the solvent was removed under reduced pressure and the crude product purified by reverse phase column chromatography with a linear gradient (25%/75% to 100%/0% methanol/water). The fractions containing the product were combined, and the compound was dried under vacuum. Yield: 36 mg (0.08 mmol, 60%). ^1^H NMR [400 MHz, (CD_3_)_2_CO]: δ 7.58–7.51 (m, 2H), 7.32–7.28 (m, 2H), 7.19–7.16 (m, 1H), 4.09 (dd, *J* = 13.3, 13.1 Hz, 2H), 3.81 (dd, *J* = 13.5, 13.2 Hz, 2H), 3.06 (dd, *J* = 13.4, 13.1 Hz, 2H), 2.07 (dd, *J* = 13.6, 13.1 Hz, 2H). ^13^C{^1^H} NMR [100 MHz, (CD_3_)_2_CO]: δ 139.2, 135.8, 131.9, 128.4, 50.7, 44.1. HR-MS (*m*/*z*): [M + H]^+^ calcd for C_10_H_14_OReS_4_, 464.9479; found, 464.9482. RP-HPLC: *t*_R_ = 15.1 min.

#### ReO(2,2′-thiodiethanethiol)(4-phenylbenzene-1-thiol) (**6**)

Re(2,2′-thiodiethanethiol)(water) trifluoromethanesulfonate (20.0 mg, 0.038 mmol) was dissolved in dry acetonitrile (10 mL), and 4-phenylbenzene-1-thiol (9.3 mg, 0.050 mmol) added. The reaction mixture was stirred at room temperature for 2 h in the dark. After this time, the solvent was removed under reduced pressure and the crude product purified by reverse phase column chromatography with a linear gradient (50%/50% to 100%/0% methanol/water). The fractions containing the product were combined, and the compound was dried under vacuum. Yield: 11 mg (0.020 mmol, 53%). ^1^H NMR [400 MHz, (CD_3_)_2_CO]: δ 7.75 (d, *J* = 7.1 Hz, 2H), 7.63 (d, *J* = 7.8 Hz, 2H), 7.58 (d, *J* = 7.8 Hz, 2H), 7.48–7.40 (m, 3H), 4.17 (dd, *J* = 13.5, 13.2 Hz, 2H), 3.93 (dd, *J* = 13.5, 13.1 Hz, 2H), 2.97 (dd, *J* = 13.3, 13.2 Hz, 2H), 2.01 (dd, *J* = 13.4, 13.2 Hz, 2H). ^13^C{^1^H} NMR [100 MHz, (CD_3_)_2_CO]: δ 141.3, 138.6, 132.7, 129.4, 128.8, 127.9, 127.6, 127.5, 51.3, 44.1. HR-MS (*m*/*z*): [M + H]^+^ calcd for C_16_H_18_OReS_4_, 540.9792; found, 540.9795. RP-HPLC: *t*_R_ = 18.9 min.

#### ReO(2,2′-thiodiethanethiol)(l-cysteine) (**7**)

This compound has been previously reported,^19^ but a different synthetic procedure was used here. Re(2,2′-thiodiethanethiol)(water) trifluoromethanesulfonate (20.0 mg, 0.038 mmol) was dissolved in dry acetonitrile (10 mL), and l-cysteine (6.0 mg, 0.050 mmol) added. The reaction mixture was stirred at room temperature for 2 h in the dark. After this time, the solvent was removed under reduced pressure and the crude product purified by reverse phase column chromatography with a linear gradient (25%/75% to 100%/0% methanol/water). The fractions containing the product were combined, and the compound was dried under vacuum. Yield: 8.1 mg (0.017 mmol, 44%). ^1^H NMR [400 MHz, (CD_3_)_2_CO]: δ 4.38 (dd, *J* = 14.1, 13.2 Hz, 2H), 4.18–4.22 (d, *J* = 13.8, 13.4 Hz, 2H), 4.01–4.04 (m, 1H), 3.24 (dd, *J* = 14.1, 13.4 Hz, 2H), 3.24–3.18 (m, 2H), 2.75–2.65 (dd, *J* = 13.9, 13.3 Hz, 2H). ^13^C{^1^H} NMR [100 MHz, (CD_3_)_2_CO]: δ 174.3, 59.6, 46.2, 50.9, 44.6. HR-MS (*m*/*z*): [M + H]^+^ calcd for C_7_H_15_NO_3_ReS_4_, 475.9489; found, 475.9490. RP-HPLC: *t*_R_ = 10.1 min.

#### ReO(2,2′-thiodiethanethiol)(glutathione) (**8**)

This compound has been previously reported,^19^ but a different synthetic procedure was used here. Re(2,2′-thiodiethanethiol)(water) trifluoromethanesulfonate (20.0 mg, 0.038 mmol) was dissolved in dry acetonitrile (10 mL), and glutathione (15.4 mg, 0.050 mmol) added. The reaction mixture was heated at 40 °C for 2 h in the dark. After this time, the solvent was removed under reduced pressure and the crude product purified by reverse phase column chromatography with a linear gradient (25%/75% to 100%/0% methanol/water). The fractions containing the product were combined, and the compound was dried under vacuum. Yield: 5.9 mg (0.009 mmol, 24%). ^1^H NMR (400 MHz, D_2_O): δ 4.18 (d, *J* = 8.2 Hz, 1H), 4.16–4.03 (m, 2H), 3.97 (dd, *J* = 13.3, 13.2 Hz, 2H), 3.84–3.73 (m, 2H), 3.72–3.61 (m, 1H), 2.97 (dd, *J* = 13.2, 13.0 Hz, 2H), 2.78–2.64 (m, 2H), 2.41 (d, *J* = 7.8 Hz, 2H), 2.11–2.01 (m, 4H). ^13^C{^1^H} NMR [100 MHz, (CD_3_)_2_CO]: δ 171.3, 169.7, 169.4, 168.6, 58.7, 55.6, 51.4, 50.7, 44.5, 44.0, 31.2, 26.3. HR-MS (*m*/*z*): [M + H]^+^ calcd for C_14_H_25_N_3_O_7_ReS_4_, 662.0129; found, 662.0131. RP-HPLC: *t*_R_ = 9.4 min.

#### *N*,*N*-Bis(2-hydroxyethyl)aniline

The compound was prepared using a reported protocol^19^ with minor modifications. Aniline (50 μL, 0.55 mmol) and propionic acid (0.5 μL) were dissolved in water (10 mL). The solution was cooled down to 0 °C and ethylene oxide (973 μL of a 1.13 M solution in methanol, 1.10 mmol) was added dropwise. The mixture was stirred at room temperature overnight. The compound was extracted with dichloromethane (3 × 20 mL) and washed with a saturated aqueous solution of sodium bicarbonate (2 × 20 mL). The solvent was removed under reduced pressure. The crude product was recrystallized in methanol. The compound was dried under vacuum. Yield: 81 mg (0.45 mmol, 82%). ^1^H NMR (500 MHz, CD_2_Cl_2_): δ 7.20 (dd, *J* = 8.2, 7.1 Hz, 2H), 6.70 (d, *J* = 8.2 Hz, 2H), 6.69 (d, *J* = 7.1 Hz, 1H), 3.81 (t, *J* = 5.0 Hz, 4H), 3.55 (t, *J* = 5.0 Hz, 4H); ^13^C{^1^H}-NMR (125 MHz, CD_2_Cl_2_): δ 148.3, 129.5, 116.9, 112.8, 61.0, 55.6; HR-MS (*m*/*z*): [M + H]^+^ calcd. for C_10_H_16_NO_2_, 182.1176; found, 182.1175.

#### *N*,*N*-Bis(2-tosylethyl)aniline

The compound was prepared using a reported protocol^50^ with minor modifications. *N*,*N*-Bis(2-hydroxyethyl)aniline (1 g, 5.5 mmol) and pyridine (4 mL) were dissolved in dichloromethane (35 mL) at 0 °C. 4-Methylbenzenesulfonyl chloride (3.2 g, 16.8 mmol) was added slowly, and the reaction was allowed to return to room temperature and stirred overnight. The reaction mixture was washed with water (3 × 20 mL) and 1 M hydrogen chloride solution in water (3 × 20 mL). The organic phase was dried over magnesium sulfate. The solvent was removed under reduced pressure and the crude product purified by silica column chromatography with a linear gradient (0%:100% - 100%:0% dichloromethane/hexane). The fractions containing the product were combined and the compound dried under vacuum. Yield: 2.3 g (4.7 mmol, 85%). ^1^H NMR (500 MHz, CD_2_Cl_2_): δ 7.67 (d, *J* = 8.3 Hz, 4H), 7.28 (d, *J* = 8.3 Hz, 4H), 7.10 (dd, *J* = 8.9, 7.3 Hz, 2H), 6.67 (dd, *J* = 7.3, 0.9 Hz, 1H), 6.38 (dd, *J* = 8.9, 0.9 Hz, 2H), 4.05 (t, *J* = 6.0 Hz, 4H), 3.51 (t, *J* = 6.0 Hz, 4H), 2.40 (s, 6H); ^13^C{^1^H}-NMR (125 MHz, CD_2_Cl_2_): δ 146.1, 145.6, 132.7, 130.2, 129.7, 128.1, 117.6, 112.2, 66.9, 50.8, 22.4; HR-MS (*m*/*z*): [M + H]^+^ calcd. for C_24_H_28_NO_6_S_2_, 490.1353; found, 490.1352.

#### *N*,*N*-Bis(2-thioethyl)aniline

The compound was prepared using a reported protocol^51^ with minor modifications. *N*,*N*-Bis(2-tosylethyl)aniline (1 g, 2.0 mmol) and thiourea (1.5 g, 20 mmol) were dissolved in ethanol (40 mL) and heated at reflux for 3 h. After this time, the solution was concentrated to ∼5 mL under reduced pressure and a saturated aqueous solution of sodium bicarbonate added (40 mL). The mixture was heated at reflux for 4 h. After this time, the solution was allowed to return to room temperature and the organic phase extracted with chloroform (3 × 50 mL). The solution was dried over magnesium sulfate and the solvent was removed under reduced pressure. The crude product was recrystallized in ethanol. The compound was dried under vacuum. Yield: 0.32 g (1.5 mmol, 76%). ^1^H NMR (500 MHz, CD_2_Cl_2_): δ 7.21 (dd, *J* = 7.8, 7.3 Hz, 2H), 6.69 (d, *J* = 7.8 Hz, 2H), 6.64 (d, *J* = 7.3 Hz, 1H), 3.52 (t, *J* = 5.6 Hz, 4H), 2.74 (t, *J* = 5.6 Hz, 4H); ^13^C{^1^H}-NMR (125 MHz, CD_2_Cl_2_): δ 146.2, 129.4, 117.0, 112.0, 54.8, 21.9; HR-MS (*m*/*z*): [M + H]^+^ calcd. for C_10_H_16_NS_2_, 214.0719; found, 214.0717.

#### ReO[*N*,*N*-bis(2-thioethyl)aniline](chloride) (**9**)

Tetrabutylammonium tetrachlorooxorhenate(V) (138 mg, 0.23 mmol) was dissolved in dry methanol (20 mL) under a nitrogen atmosphere. A solution of *N*,*N*-bis(2-thioethyl)aniline (50 mg, 0.23 mmol) in dry chloroform (5 mL) was added dropwise. The mixture was stirred at room temperature overnight. After this time, the solvent was removed under reduced pressure and the crude product purified by reverse phase column chromatography with a linear gradient (25%/75% to 100%/0% methanol/water). The fractions containing the product were combined, and the compound was dried under vacuum. Yield: 63 mg (0.14 mmol, 61%). ^1^H NMR [500 MHz, (CD_3_)_2_CO]: δ 8.16 (d, *J* = 8.3 Hz, 2H), 7.59 (dd, *J* = 8.3, 7.1 Hz, 2H), 7.55 (d, *J* = 7.1 Hz, 1H), 3.44 (dd, *J* = 13.4, 13.2 Hz, 2H), 1.79 (dd, *J* = 13.5, 13.3 Hz, 2H), 1.43 (dd, *J* = 13.4, 13.1 Hz, 2H), 0.97 (dd, *J* = 13.5, 13.2 Hz, 2H). ^13^C{^1^H} NMR [125 MHz, (CD_3_)_2_CO]: δ 143.8, 130.2, 129.9, 122.1, 58.6, 23.8. HR-MS (*m*/*z*): [M – Cl]^+^ calcd for C_10_H_13_NOReS_2_, 413.9987; found, 413.9984. RP-HPLC: *t*_R_ = 17.3 min.

#### *N*,*N*-Bis(2-hydroxyethyl)-4-bromoaniline

The compound was prepared using a reported protocol^52^ with minor modifications. *N*,*N*-Bis(2-hydroxyethyl)aniline (500 mg, 2.8 mmol) and *N*-bromosuccinimide (534 mg, 3.0 mmol) were dissolved in dichloromethane (30 mL), and the mixture was stirred at room temperature overnight. The solution was washed with a saturated aqueous solution of sodium bicarbonate (3 × 20 mL) and dried over magnesium sulfate. The solvent was removed under reduced pressure, and the crude product purified by silica column chromatography with a linear gradient (0%/100% to 100%/0% dichloromethane/hexane). The fractions containing the product were combined, and the compound was dried under vacuum. Yield: 640 mg (2.5 mmol, 88%). ^1^H NMR (500 MHz, CD_2_Cl_2_): δ 7.27 (d, *J* = 9.1 Hz, 2H), 6.56 (d, *J* = 9.1 Hz, 2H), 3.77 (t, *J* = 4.9 Hz, 4H), 3.51 (t, *J* = 4.9 Hz, 4H). ^13^C{^1^H} NMR (125 MHz, CD_2_Cl_2_): δ 147.3, 132.1, 114.4, 108.5, 60.7, 55.6. HR-MS (*m*/*z*): [M + H]^+^ calcd for C_10_H_15_BrNO_2_, 260.0281; found, 260.0276.

#### *N*,*N*-Bis(2-tosylethyl)-4-bromoaniline

The compound was prepared using a reported protocol^53^ with minor modifications. *N*,*N*-Bis(2-hydroxyethyl)-4-bromoaniline (500 mg, 1.9 mmol) and pyridine (4 mL) were dissolved in dichloromethane (35 mL) at 0 °C. 4-Methylbenzenesulfonyl chloride (1.1 g, 5.8 mmol) was added slowly, and the reaction mixture was allowed to return to room temperature and stirred overnight. The reaction mixture was washed with water (3 × 20 mL) and a 1 M hydrogen chloride solution in water (3 × 20 mL). The organic phase was dried over magnesium sulfate. The solvent was removed under reduced pressure, and the crude product purified by silica column chromatography with a linear gradient (0%/100% to 100%/0% dichloromethane/hexane). The fractions containing the product were combined, and the compound was dried under vacuum. Yield: 852 mg (1.5 mmol, 79%). ^1^H NMR (500 MHz, CD_2_Cl_2_): δ 7.53 (d, *J* = 8.2 Hz, 4H), 7.36 (d, *J* = 8.2 Hz, 4H), 7.19 (d, *J* = 9.0 Hz, 2H), 6.69 (d, *J* = 9.0 Hz, 2H), 3.88 (t, *J* = 5.9 Hz, 4H), 3.55 (t, *J* = 5.9 Hz, 4H), 2.40 (s, 6H). ^13^C{^1^H} NMR (125 MHz, CD_2_Cl_2_): δ 145.5, 145.2, 132.7, 131.3, 130.2, 128.1, 114.0, 109.9, 66.6, 50.7, 22.0. HR-MS (*m*/*z*): [M + H]^+^ calcd for C_24_H_27_BrNO_6_S_2_, 568.0458; found, 568.0462.

#### *N*,*N*-Bis(2-thioethyl)-4-bromoaniline

*N*,*N*-Bis(2-tosylethyl)-4-bromoaniline (250 mg, 0.4 mmol) and thiourea (334 mg, 4.4 mmol) were dissolved in ethanol (20 mL) and heated at reflux for 3 h. After this time, the solution was concentrated to ∼5 mL under reduced pressure and a saturated aqueous solution of sodium bicarbonate added (20 mL). The mixture was heated at reflux for 4 h. After this time, the solution was allowed to return to room temperature and the organic phase extracted with chloroform (3 × 50 mL). The solution was dried over magnesium sulfate, and the solvent was removed under reduced pressure. The crude product was recrystallized in ethanol. The compound was dried under vacuum. Yield: 82 mg (0.3 mmol, 64%). ^1^H NMR (500 MHz, CD_2_Cl_2_): δ 7.22 (d, *J* = 7.9 Hz, 2H), 6.68 (d, *J* = 7.9 Hz, 2H), 3.71 (t, *J* = 5.8 Hz, 4H), 2.81 (t, *J* = 5.8 Hz, 4H). ^13^C{^1^H} NMR (125 MHz, CD_2_Cl_2_): δ 147.9, 129.1, 116.5, 112.4, 59.1, 24.3. HR-MS (*m*/*z*): [M + H]^+^ calcd for C_10_H_15_BrNS_2_, 291.9829; found, 291.9826.

#### ReO[*N*,*N*-bis(2-thioethyl)-4-bromoaniline](chloride) (**10**)

Tetrabutylammonium tetrachlorooxorhenate(V) (100 mg, 0.17 mmol) was dissolved in dry methanol (20 mL) under a nitrogen atmosphere. A solution of *N*,*N*-bis(2-thioethyl)-4-bromoaniline (50 mg, 0.17 mmol) in dry chloroform (5 mL) was added dropwise. The mixture was stirred at room temperature overnight. After this time, the solvent was removed under reduced pressure and the crude product purified by reverse phase column chromatography with a linear gradient (25%/75% to 100%/0% methanol/water). The fractions containing the product were combined, and the compound was dried under vacuum. Yield: 79 mg (0.15 mmol, 88%). ^1^H NMR [500 MHz, (CD_3_)_2_CO]: δ 8.12 (d, *J* = 8.1 Hz, 2H), 7.19 (d, *J* = 8.1 Hz, 2H), 3.52 (dd, *J* = 13.3, 13.2 Hz, 2H), 1.80 (dd, *J* = 13.3, 13.2 Hz, 2H), 1.46 (dd, *J* = 13.3, 13.2 Hz, 2H), 0.98 (dd, *J* = 13.3, 13.2 Hz, 2H). ^13^C{^1^H} NMR [125 MHz, (CD_3_)_2_CO]: δ 143.3, 129.7, 121.4, 118.6, 58.8, 24.1. HR-MS (*m*/*z*): [M – Cl]^+^ calcd for C_10_H_12_BrNOReS_2_, 491.9101; found, 491.9098.

#### *N*,*N*-Bis(2-hydroxyethyl)-4-boronic Acid Pinacol Ester Aniline

The compound was prepared using a reported protocol^54^ with minor modifications. *N*,*N*-Bis(2-hydroxyethyl)-4-bromoaniline (500 mg, 1.9 mmol), bis(pinacolato)diboron (965 mg, 3.8 mmol), and potassium acetate (559 mg, 5.7 mmol) were dissolved in dry dioxane (25 mL), and the solution was degassed. [1,1′-Bis(diphenylphosphino)ferrocene]palladium(II) dichloride (146 mg, 0.2 mmol) was added, and the mixture refluxed overnight under a nitrogen atmosphere. After this time, ethyl acetate (30 mL) and water (30 mL) were added to the mixture. The compound was extracted with ethyl acetate (3 × 20 mL) and washed with a saturated aqueous solution of sodium bicarbonate (2 × 20 mL) and brine (2 × 20 mL). The solvent was removed under reduced pressure, and the crude product purified by silica column chromatography with a linear gradient (0%/100% to 100%/0% ethyl acetate/hexane). The fractions containing the product were combined, and the compound was dried under vacuum. Yield: 123 mg (0.4 mmol, 21%). ^1^H NMR (500 MHz, CD_2_Cl_2_): δ 7.57 (d, *J* = 8.7 Hz, 2H), 6.65 (d, *J* = 8.7 Hz, 2H), 3.81 (t, *J* = 4.9 Hz, 4H), 3.59 (t, *J* = 4.9 Hz, 4H), 1.29 (s, 12H). ^13^C{^1^H} NMR (125 MHz, CD_2_Cl_2_): δ 144.8, 136.1, 129.2, 111.4, 83.2, 60.6, 55.0, 24.6. HR-MS (*m*/*z*): [M + H]^+^ calcd for C_16_H_27_BNO_4_, 308.2031; found, 308.2028.

#### *N*,*N*-Bis(2-tosylethyl)-4-boronic Acid Pinacol Ester Aniline

*N*,*N*-Bis(2-hydroxyethyl)-4-boronic acid pinacol ester aniline (100 mg, 0.33 mmol) and pyridine (2 mL) were dissolved in dichloromethane (20 mL) at 0 °C. 4-Methylbenzenesulfonyl chloride (191 mg, 1.00 mmol) was added slowly, and the reaction mixture was allowed to return to room temperature and stirred overnight. The reaction mixture was washed with water (3 × 20 mL) and a 1 M hydrogen chloride solution in water (3 × 20 mL). The organic phase was dried over magnesium sulfate. The solvent was removed under reduced pressure, and the crude product purified by silica column chromatography with a linear gradient (0%/100% to 100%/0% dichloromethane/hexane). The fractions containing the product were combined, and the compound was dried under vacuum. Yield: 160 mg (0.26 mmol, 78%). ^1^H NMR (500 MHz, CD_2_Cl_2_): δ 7.56 (d, *J* = 8.1 Hz, 4H), 7.29 (d, *J* = 8.1 Hz, 4H), 7.11 (d, *J* = 8.9 Hz, 2H), 6.53 (d, *J* = 8.9 Hz, 2H), 3.73 (t, *J* = 5.0 Hz, 4H), 3.45 (t, *J* = 5.0 Hz, 4H), 2.38 (s, 6H), 1.23 (s, 12H). ^13^C{^1^H} NMR (125 MHz, CD_2_Cl_2_): δ 146.1, 145.8, 132.8, 130.9, 130.6, 129.7, 113.8, 110.5, 81.4, 66.3, 51.2, 24.7, 22.6. HR-MS (*m*/*z*): [M + H]^+^ calcd for C_30_H_39_BNO_8_S_2_, 616.2210; found, 616.2208.

#### *N*,*N*-Bis(2-thioethyl)-4-boronic Acid Pinacol Ester Aniline

*N*,*N*-Bis(2-tosylethyl)-4-boronic acid pinacol ester aniline (200 mg, 0.32 mmol), thiolactic acid (58 μL, 0.66 mmol), and triethylamine (92 μL, 0.66 mmol) were dissolved in dry tetrahydrofuran (30 mL). The mixture was stirred at room temperature overnight. The solvent was removed under reduced pressure, and the residue redissolved in methanol (30 mL). A 6 M hydrogen chloride solution in water (10 mL) was added, and the mixture stirred at room temperature overnight. The solvent was removed under reduced pressure, and the crude product purified by silica column chromatography with a linear gradient (0%/100% to 100%/0% dichloromethane/hexane). The fractions containing the product were combined, and the compound was dried under vacuum. Yield: 14 mg (0.04 mmol, 13%). ^1^H NMR (500 MHz, CD_2_Cl_2_): δ 7.51 (d, *J* = 8.8 Hz, 2H), 6.71 (d, *J* = 8.8 Hz, 2H), 3.76 (t, *J* = 5.1 Hz, 4H), 2.87 (t, *J* = 5.1 Hz, 4H), 1.30 (s, 12H). ^13^C{^1^H} NMR (125 MHz, CD_2_Cl_2_): δ 145.7, 133.3, 123.4, 112.6, 82.9, 59.6, 24.1, 23.5. HR-MS (*m*/*z*): [M + H]^+^ calcd for C_16_H_27_BNO_2_S_2_, 340.1573; found, 340.1571.

#### ReO[*N*,*N*-bis(2-thioethyl)-4-boronic acid pinacol ester aniline](chloride) (**11**)

Tetrabutylammonium tetrachlorooxorhenate(V) (43 mg, 0.07 mmol) was dissolved in dry methanol (15 mL) under a nitrogen atmosphere. A solution of *N*,*N*-bis(2-thioethyl)-4-boronic acid pinacol ester aniline (25 mg, 0.07 mmol) in dry chloroform (3 mL) was added dropwise. The mixture was stirred at room temperature overnight. After this time, the solvent was removed under reduced pressure and the crude product purified by reverse phase column chromatography with a linear gradient (25%/75% to 100%/0% methanol/water). The fractions containing the product were combined, and the compound was dried under vacuum. Yield: 26 mg (0.04 mmol, 61%). ^1^H NMR [500 MHz, (CD_3_)_2_CO]: δ 8.11 (d, *J* = 8.6 Hz, 2H), 7.42 (d, *J* = 8.6 Hz, 2H), 3.62 (dd, *J* = 13.4, 13.2 Hz, 2H), 1.91 (dd, *J* = 13.4, 13.2 Hz, 2H), 1.51 (dd, *J* = 13.4, 13.2 Hz, 2H), 1.31 (s, 12H), 0.96 (dd, *J* = 13.4, 13.2 Hz, 2H). ^13^C{^1^H} NMR [125 MHz, (CD_3_)_2_CO]: δ 145.1, 130.8, 128.9, 116.7, 82.7, 56.2, 24.1, 23.7. HR-MS (*m*/*z*): [M – Cl]^+^ calcd for C_16_H_24_BNO_3_ReS_2_, 540.0846; found, 540.0844.

#### *N*,*N*-Bis(2-hydroxyethyl)-4-carboxlic Acid Ethyl Ester Aniline

Ethyl-4-aminobenzoate (500 mg, 3.0 mmol) and propionic acid (5 μL) were dissolved in water (50 mL). The solution was cooled to 0 °C, and ethylene oxide (5.8 mL of a 1.13 M solution in methanol, 6.60 mmol) was added dropwise. The mixture was stirred at room temperature overnight. The compound was extracted with dichloromethane (3 × 30 mL) and washed with a saturated aqueous solution of sodium bicarbonate (2 × 20 mL). The solvent was removed under reduced pressure. The crude product was recrystallized in methanol. The compound was dried under vacuum. Yield: 516 mg (2.0 mmol, 68%). ^1^H NMR (500 MHz, CD_2_Cl_2_): δ 7.78 (d, *J* = 8.1 Hz, 2H), 6.91 (d, *J* = 8.1 Hz, 2H), 4.33 (q, *J* = 7.2 Hz, 2H), 4.09 (t, *J* = 5.3 Hz, 4H), 3.67 (t, *J* = 5.3 Hz, 4H), 1.37 (t, *J* = 7.2 Hz, 3H). ^13^C{^1^H} NMR (125 MHz, CD_2_Cl_2_): δ 165.7, 151.6, 129.1, 117.3, 111.6, 62.4, 61.2, 59.7, 15.2. HR-MS (*m*/*z*): [M + H]^+^ calcd for C_13_H_20_NO_4_, 254.1392; found, 254.1391.

#### *N*,*N*-Bis(2-tosylethyl)-4-carboxlic Acid Ethyl Ester Aniline

*N*,*N*-Bis(2-hydroxyethyl)-4-carboxlic acid aniline (100 mg, 0.40 mmol) and pyridine (2 mL) were dissolved in dichloromethane (20 mL) at 0 °C. 4-Methylbenzenesulfonyl chloride (229 mg, 1.20 mmol) was added slowly, and the reaction mixture was allowed to return to room temperature and stirred overnight. The reaction mixture was washed with water (3 × 20 mL) and a 1 M hydrogen chloride solution in water (3 × 20 mL). The organic phase was dried over magnesium sulfate. The solvent was removed under reduced pressure, and the crude product purified by silica column chromatography with a linear gradient (0%/100% to 100%/0% dichloromethane/hexane). The fractions containing the product were combined, and the compound was dried under vacuum. Yield: 139 mg (0.25 mmol, 62%). ^1^H NMR (500 MHz, CD_2_Cl_2_): δ 7.76 (d, *J* = 8.0 Hz, 2H), 7.63 (d, *J* = 8.2 Hz, 4H), 7.46 (d, *J* = 8.2 Hz, 4H), 6.90 (d, *J* = 8.0 Hz, 2H), 4.34 (q, *J* = 7.2 Hz, 2H), 4.11 (t, *J* = 4.9 Hz, 4H), 3.71 (t, *J* = 4.9 Hz, 4H), 2.37 (s, 6H), 1.39 (t, *J* = 7.2 Hz, 3H). ^13^C{^1^H} NMR (125 MHz, CD_2_Cl_2_): δ 163.5, 149.4, 145.7, 132.6, 129.6, 128.7, 116.7, 113.4, 110.9, 63.3, 61.4, 58.5, 23.5, 15.4. HR-MS (*m*/*z*): [M + H]^+^ calcd for C_27_H_32_NO_8_S_2_, 562.1562; found, 562.1534.

#### *N*,*N*-Bis(2-thioethyl)-4-carboxlic Acid Aniline

*N*,*N*-Bis(2-tosylethyl)-4-carboxlic acid ethyl ester aniline (100 mg, 0.18 mmol) and thiourea (137 mg, 1.8 mmol) were dissolved in ethanol (20 mL) and heated at reflux for 4 h. After this time, the solution was concentrated to ∼5 mL under reduced pressure and an aqueous 1 M sodium hydroxide solution added (30 mL). The mixture was stirred at room temperature overnight. After this time, an aqueous 1 M hydrogen chloride solution was added until the pH reached <1. The compound was extracted with chloroform (3 × 30 mL) and washed with brine (2 × 20 mL). The solution was dried over magnesium sulfate, and the solvent was removed under reduced pressure. The crude product was recrystallized in methanol. The compound was dried under vacuum. Yield: 19 mg (0.08 mmol, 42%). ^1^H NMR (500 MHz, CD_2_Cl_2_): δ 7.68 (d, *J* = 8.2 Hz, 2H), 6.96 (d, *J* = 8.2 Hz, 2H), 3.62 (t, *J* = 5.4 Hz, 4H), 2.81 (t, *J* = 5.4 Hz, 4H). ^13^C{^1^H} NMR (125 MHz, CD_2_Cl_2_): δ 165.2, 151.4, 130.7, 119.6, 111.0, 59.4, 23.2. HR-MS (*m*/*z*): [M – H]^−^ calcd for C_11_H_14_NO_2_S_2_, 256.0466; found, 256.0463.

#### ReO[*N*,*N*-bis(2-thioethyl)-4-carboxlic acid aniline](chloride) (**12**)

Tetrabutylammonium tetrachlorooxorhenate(V) (59 mg, 0.10 mmol) was dissolved in dry methanol (15 mL) under a nitrogen atmosphere. A solution of *N*,*N*-bis(2-thioethyl)-4-carboxlic acid aniline (25 mg, 0.10 mmol) in dry chloroform (3 mL) was added dropwise. The mixture was stirred at room temperature overnight. After this time, the solvent was removed under reduced pressure and the crude product purified by reverse phase column chromatography with a linear gradient (25%/75% to 100%/0% methanol/water). The fractions containing the product were combined, and the compound was dried under vacuum. Yield: 35 mg (0.07 mmol, 72%). ^1^H NMR [500 MHz, (CD_3_)_2_CO]: δ 8.21 (d, *J* = 8.2 Hz, 2H), 7.24 (d, *J* = 8.2 Hz, 2H), 3.32 (dd, *J* = 13.4, 13.2 Hz, 2H), 1.91 (dd, *J* = 13.4, 13.2 Hz, 2H), 1.52 (dd, *J* = 13.4, 13.2 Hz, 2H), 0.99 (dd, *J* = 13.4, 13.2 Hz, 2H). ^13^C{^1^H} NMR [125 MHz, (CD_3_)_2_CO]: δ 164.7, 149.6, 130.0, 121.2, 111.7, 59.1, 24.3. HR-MS (*m*/*z*): [M – Cl]^+^ calcd for C_11_H_13_NO_3_ReS_2_, 457.9891; found, 457.9889.

#### *N*,*N*-Bis(2-hydroxypropyl)aniline

This compound has been previously reported,^55^ but a different synthetic procedure was used here. Aniline (200 μL, 2.2 mmol) and propionic acid (2 μL) were dissolved in water (20 mL). The solution was cooled to 0 °C, and 1,2-propylene oxide (300 μL, 4.4 mmol) was added dropwise. The mixture was stirred at room temperature overnight. The compound was extracted with dichloromethane (3 × 20 mL) and washed with a saturated aqueous solution of sodium bicarbonate (2 × 20 mL). The solvent was removed under reduced pressure. The crude product was recrystallized in methanol. The compound was dried under vacuum. The stereoisomers obtained during the synthesis were not separated. Yield: 671 mg (3.2 mmol, 73%). ^1^H NMR (500 MHz, CD_2_Cl_2_): δ 7.21–7.15 (m, 2H), 6.78 (d, *J* = 8.8 Hz, 1H), 6.71–6.67 (m, 1H), 6.56 (d, *J* = 7.9 Hz, 1H), 4.16–4.04 (m, 1H), 3.64 (d, *J* = 15.2 Hz, 1H), 3.37 (d, *J* = 14.8 Hz, 1H), 3.19–3.12 (m, 2H), 3.00–2.92 (m, 1H), 1.19–1.14 (m, 6H). ^13^C{^1^H} NMR (125 MHz, CD_2_Cl_2_): δ 149.2, 148.1, 129.1, 129.0, 117.1, 116.5, 113.8, 112.2, 66.0, 64.9, 62.5, 59.7, 20.1. HR-MS (*m*/*z*): [M + H]^+^ calcd for C_12_H_20_NO_2_, 210.1494; found, 210.1493.

#### *N*,*N*-Bis(2-tosylpropyl)aniline

*N*,*N*-Bis(2-hydroxypropyl)aniline (200 mg, 0.96 mmol) and pyridine (2 mL) were dissolved in dichloromethane (20 mL) at 0 °C. 4-Methylbenzenesulfonyl chloride (590 mg, 2.88 mmol) was added slowly, and the reaction mixture was allowed to return to room temperature and stirred overnight. The reaction mixture was washed with water (3 × 20 mL) and a 1 M hydrogen chloride solution in water (3 × 20 mL). The organic phase was dried over magnesium sulfate. The solvent was removed under reduced pressure, and the crude product purified by silica column chromatography with a linear gradient (0%/100% to 100%/0% dichloromethane/hexane). The fractions containing the product were combined, and the compound was dried under vacuum. The stereoisomers obtained during the synthesis were not separated. Yield: 308 mg (0.60 mmol, 62%). ^1^H NMR (500 MHz, CD_2_Cl_2_): δ 7.72–7.63 (m, 4H), 7.33–7.25 (m, 4H), 7.23–7.12 (m, 2H), 6.76 (d, *J* = 8.3 Hz, 1H), 6.73–6.62 (m, 1H), 6.61–6.52 (m, 1H), 4.38–4.22 (m, 1H), 4.05–3.93 (m, 2H), 3.62 (d, *J* = 15.0 Hz, 1H), 3.41 (d, *J* = 15.0 Hz, 1H), 3.32–3.21 (m, 1H), 2.40 (s, 6H), 1.19–1.12 (m, 6H). ^13^C{^1^H} NMR (125 MHz, CD_2_Cl_2_): δ 147.6, 146.1, 145.3, 145.1, 140.1, 139.5, 131.4, 131.2, 130.3, 129.9, 128.2, 128.1, 119.6, 119.4, 111.7, 110.5, 74.2, 72.1, 62.4, 61.3, 22.4, 20.2. HR-MS (*m*/*z*): [M + H]^+^ calcd for C_26_H_32_NO_6_S_2_, 518.1671; found, 518.1668.

#### *N*,*N*-Bis(2-thiopropyl)aniline

*N*,*N*-Bis(2-tosylpropyl)aniline (100 mg, 0.19 mmol) and thiourea (145 mg, 1.90 mmol) were dissolved in ethanol (15 mL) and heated at reflux for 3 h. After this time, the solution was concentrated to ∼5 mL under reduced pressure and a saturated aqueous solution of sodium bicarbonate added (10 mL). The mixture was heated at reflux for 4 h. After this time, the solution was allowed to return to room temperature and the organic phase extracted with chloroform (3 × 20 mL). The solution was dried over magnesium sulfate, and the solvent was removed under reduced pressure. The crude product was recrystallized in methanol. The compound was dried under vacuum. The stereoisomers obtained from the synthesis were not separated. Yield: 29 mg (0.12 mmol, 64%). ^1^H NMR (500 MHz, CD_2_Cl_2_): δ 7.25–7.18 (m, 2H), 6.81–6.75 (m, 1H), 6.72–6.66 (m, 1H), 6.62 (d, *J* = 8.2 Hz, 1H), 3.71–3.48 (m, 4H), 2.72–2.43 (m, 2H), 1.52–1.41 (m, 6H). ^13^C{^1^H} NMR (125 MHz, CD_2_Cl_2_): δ 149.5, 1487.9, 129.3, 129.2, 117.4, 116.8, 114.1, 112.6, 56.7, 56.2, 28.9, 27.3, 22.4. HR-MS (*m*/*z*): [M + H]^+^ calcd for C_12_H_20_NS_2_, 242.1037; found, 242.1035.

#### ReO[*N*,*N*-bis(2-thiopropyl)aniline](chloride) (**13**)

Tetrabutylammonium tetrachlorooxorhenate(V) (61 mg, 0.10 mmol) was dissolved in dry methanol (10 mL) under a nitrogen atmosphere. A solution of *N*,*N*-bis(2-thiopropyl)aniline (25 mg, 0.10 mmol) in dry chloroform (5 mL) was added dropwise. The mixture was stirred at room temperature overnight. After this time, the solvent was removed under reduced pressure and the crude product purified by reverse phase column chromatography with a linear gradient (25%/75% to 100%/0% methanol/water). The fractions containing the product were combined, and the compound was dried under vacuum. The stereoisomers obtained from the synthesis were not separated. Yield: 26 mg (0.05 mmol, 52%). ^1^H NMR [500 MHz, (CD_3_)_2_CO]: δ 8.23–8.11 (m, 2H), 7.61–7.41 (m, 3H), 3.51–3.45 (m, 1H), 3.37–3.28 (m, 1H), 1.83–1.79 (m, 1H), 1.76–1.75 (m, 1H), 1.42–1.38 (m, 6H), 1.01–0.98 (m, 1H), 0.97–0.94 (m, 1H). ^13^C{^1^H} NMR [125 MHz, (CD_3_)_2_CO]: δ 143.7, 143.5, 130.6, 130.2, 129.7, 129.5, 122.1, 121.5, 59.1, 58.7, 24.1, 23.7, 22.6, 21.8. HR-MS (*m*/*z*): [M – Cl]^+^ calcd for C_12_H_17_NOReS_2_, 442.0305; found, 442.0307.

#### *N*,*N*-Bis(2-hydroxyethyl)-4-(prop-2-yn-1-yloxy)aniline

*N*,*N*-Bis(2-hydroxyethyl)-4-bromoaniline (200 mg, 0.8 mmol), sodium *tert*-butoxide (73 mg, 0.8 mmol), 2-propyn-1-amine (49 μL, 0.8 mmol), and [1,1′-bis(diphenylphosphino)ferrocene]dichloropalladium(II) (4 mg) were suspended in dioxane (20 mL) and heated to 100 °C overnight under a nitrogen atmosphere. After this time, the solvent was removed under reduced pressure and the crude product purified by reverse phase column chromatography with a linear gradient (10%/90% to 100%/0% methanol/water). The fractions containing the product were combined, and the compound was dried under vacuum. Yield: 11 mg (0.05 mmol, 6%). ^1^H NMR [400 MHz, (CD_3_)_2_CO]: δ 7.10 (d, *J* = 7.9 Hz, 2H), 6.68 (d, *J* = 7.9 Hz, 2H), 3.95 (d, *J* = 2.7 Hz, 2H), 3.68 (t, *J* = 5.9 Hz, 4H), 3.49 (t, *J* = 5.9 Hz, 4H), 2.57 (t, *J* = 2.7 Hz, 1H). ^13^C{^1^H} NMR [100 MHz, (CD_3_)_2_CO]: δ 148.3, 129.0, 115.6, 111.8, 81.9, 70.9, 59.2, 54.2, 39.5. MS (*m*/*z*): [M – H]^−^ calcd for C_13_H_17_N_2_O_2_, 233.1; found, 233.6.

#### *N*,*N*-Bis(2-thioethyl)-4-(prop-2-yn-1-yloxy)aniline

*N*,*N*-Bis(2-hydroxyethyl)-4-(prop-2-yn-1-yloxy)aniline (20 mg, 0.09 mmol) and pyridine (0.5 mL) were dissolved in dichloromethane (10 mL) at 0 °C. 4-Methylbenzenesulfonyl chloride (34 mg, 0.18 mmol) was added slowly, and the reaction mixture was allowed to return to room temperature and stirred overnight. The reaction mixture was washed with water (3 × 10 mL) and a 1 M hydrogen chloride solution in water (3 × 10 mL). The organic phase was dried over magnesium sulfate. The solvent was removed under reduced pressure, and the crude product purified by reverse phase column chromatography with a linear gradient (10%/90% to 100%/0% methanol/water). The fractions containing the product were combined, and the compound was dried under vacuum. The obtained solid and thiourea (3 mg, 0.04 mmol) were dissolved in ethanol (5 mL) and heated at reflux for 3 h. After this time, the solution was concentrated to ∼1 mL under reduced pressure and a saturated aqueous solution of sodium bicarbonate added (5 mL). The mixture was heated at reflux for 4 h. After this time, the solution was allowed to return to room temperature and the organic phase extracted with chloroform (3 × 10 mL). The solution was dried over magnesium sulfate, and the solvent was removed under reduced pressure. The crude product was recrystallized in ethanol. The compound was dried under vacuum. Yield: 13 mg (0.05 mmol, 57%). ^1^H NMR (400 MHz, CD_2_Cl_2_): δ 7.25 (d, *J* = 9.0 Hz, 2H), 6.54 (d, *J* = 9.0 Hz, 2H), 3.74 (m, 4H), 3.49 (m, 4H), 3.33 (d, *J* = 2.4 Hz, 2H), 2.24 (t, *J* = 2.4 Hz, 1H). ^13^C{^1^H} NMR (100 MHz, CD_2_Cl_2_): δ 147.1, 131.8, 114.1, 108.1, 84.8, 70.1, 60.2, 55.3, 31.1. MS (*m*/*z*): [M – H]^+^ calcd for C_13_H_19_N_2_S_2_, 267.1; found, 267.3.

#### ReO[*N*,*N*-bis(2-thioethyl)-4-(prop-2-yn-1-yloxy)aniline](chloride) (**14**)

Tetrabutylammonium tetrachlorooxorhenate(V) (12 mg, 0.02 mmol) was dissolved in dry methanol (5 mL) under a nitrogen atmosphere. A solution of *N*,*N*-bis(2-thioethyl)-4-(prop-2-yn-1-yloxy)aniline (5.5 mg, 0.02 mmol) in dry chloroform (2 mL) was added dropwise. The mixture was stirred at room temperature overnight. After this time, the solvent was removed under reduced pressure and the crude product purified by reverse phase column chromatography with a linear gradient (25%/75% to 100%/0% methanol/water). The fractions containing the product were combined, and the compound was dried under vacuum. Yield: 7 mg (0.01 mmol, 33%). ^1^H NMR [500 MHz, (CD_3_)_2_CO]: δ 8.28 (d, *J* = 8.3 Hz, 2H), 7.62 (d, *J* = 8.3 Hz, 2H), 3.48 (dd, *J* = 13.3, 13.2 Hz, 2H), 3.27 (d, *J* = 2.6 Hz, 2H), 2.29 (t, *J* = 2.6 Hz, 1H), 1.83–1.76 (m, 2H), 1.42 (dd, *J* = 13.3, 13.2 Hz, 2H), 0.91 (m, 2H). ^13^C{^1^H} NMR [125 MHz, (CD_3_)_2_CO]: δ 144.1, 130.4, 120.9, 119.2, 84.8, 72.6, 59.1, 54.8, 37.6. HR-MS (*m*/*z*): [M – Cl]^+^ calcd for C_13_H_16_N_2_OReS_2_, 467.0262; found, 442.0263. RP-HPLC: *t*_R_ = 17.7 min.

#### Aqueous Solubility Tests

The aqueous solubility of the metal complexes was assessed by dynamic light scattering. The metal complexes were dissolved in DMSO at a concentration of 10 mM. The stock solutions were diluted with phosphate-buffered saline (PBS) buffer to a dilution of 2% DMSO and a compound concentration of 200 μM. The resulting solutions were analyzed by dynamic light scattering (DLS) using a Malvern Instruments Zetasizer Nano apparatus. All metal complex solutions remained clear and did not show any precipitation. Zinc oxide, which readily precipitates in an aqueous solution, was used as a positive control.

#### Aqueous Stability

The stability of compound **3** was assessed by HPLC analysis. The compound was dissolved in phosphate-buffered saline [2% (v/v) DMSO] to a concentration of 0.1 mg/mL and incubated at 37 °C for 48 h in the dark. After this time, the solution was analyzed by HPLC: Agilent 1200 series degasser and pump system with an Agilent Ecplise XDB-C18 (5 μm, 150 mm × 4.6 mm) column. The solvents (HPLC grade) were Millipore water (solvent A) and acetonitrile (solvent B). The following solvent gradient was used: 0–3 min, isocratic 95% A (5% B); 3–17 min, linear gradient from 95% A (5% B) to 0% A (100% B); 17–25 min, isocratic 0% A (100% B).

#### Interactions with Amino Acids

Metal complexes (0.1 mg/mL) were mixed in a 1:1 molar ratio with amino acids with cationic (*tert*-butoxycarbonyl-l-arginine methyl ester and *tert*-butoxycarbonyl-l-histidine methyl ester), anionic (*tert*-butoxycarbonyl-l-aspartic acid methyl ester), polar (*tert*-butoxycarbonyl-l-serine methyl ester and *tert*-butoxycarbonyl-l-asparagine methyl ester), or sulfur-containing (*tert*-butoxycarbonyl-l-cysteine methyl ester and *tert*-butoxycarbonyl-l-methionine methyl ester) side chains in water [2% (v/v) dimethyl sulfoxide]. The pH of the solution was adjusted to 7.4, and the mixture incubated at 37 °C for 24 h in the dark. After this time, the solution was analyzed by HPLC: Agilent 1200 series degasser and pump system with with an Agilent Ecplise XDB-C18 (5 μm, 150 mm × 4.6 mm) column. The solvents (HPLC grade) were Millipore water (solvent A) and acetonitrile (solvent B). The following solvent gradient was used: 0–3 min, isocratic 95% A (5% B); 3–17 min, linear gradient from 95% A (5% B) to 0% A (100% B); 17–25 min, isocratic 0% A (100% B).

#### Binding to Cysteine

The rate of the binding of compounds **1**–**5** to cysteine was determined by HPLC analysis. The compound was dissolved in water [2% (v/v) DMSO] to a concentration of 0.1 mg/mL and incubated with equimolar amounts of *tert*-butoxycarbonyl-l-cysteine methyl ester. The pH of the solution was adjusted to 7.4, and the mixture incubated for various time intervals at 37 °C in the dark. After this time, the solution was analyzed by HPLC: Agilent 1200 series degasser and pump system with with an Agilent Ecplise XDB-C18 (5 μm, 150 mm × 4.6 mm) column. The solvents (HPLC grade) were Millipore water (solvent A) and acetonitrile (solvent B). The following solvent gradient was used: 0–3 min, isocratic 75% A (25% B); 3–13 min, linear gradient from 75% A (25% B) to 0% A (100% B); 13–15 min, isocratic 0% A (100% B). The peaks in the chromatograms were integrated to determine the amount of hydrolysis of the metal complex. The reaction rate was calculated as the slope of the plot of the time in dependence concentration of starting material, based on the following equation (where *c* is the concentration, *k* the reaction rate, and *t* the time):
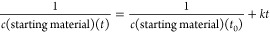


#### Electrospray Ionization Time-of-Flight Mass Spectrometry

Protein samples (0.5 μg/μL) were incubated with a Re(V) complex [50 μM, 2% (v/v) DMSO] for 2 h at room temperature with slow shaking in the dark. After this time, the protein/inhibitor mixture was analyzed by liquid chromatography electrospray ionization time-of-flight mass spectrometry (ESI-TOF-MS) using an Agilent 6230 time-of-flight mass spectrometer with a jet stream electrospray ionization source. The chromatographic separation was performed at room temperature on a Phenomenex Aeris widepore XB-C18 column [2.1 mm (inside diameter) × 50 mm (length), 3.6 μm particle size] using HPLC grade water with 0.1% trifluoroacetic acid and HPLC grade acetonitrile with 0.1% trifluoroacetic acid as mobile phases. The measured molecular weights were in agreement with the information provided by the commercial supplier: ∼34 kDa for 3CL^pro^ and ∼36.7 kDa for PL^pro^.

#### Protein Digestion and Electrospray Ionization Mass Spectrometry

The covalent binding sites of the metal complex were identified by protein digestion mass spectrometric analysis using a slight modification to a reported protocol.^56^ Protein samples (1 μg/μL, 20 μL) were incubated with the Re(V) complex [50 μM, 2% (v/v) DMSO] for 4 h at room temperature with slow shaking in the dark. After this time, the modified protein was isolated by SDS–PAGE. The band of the protein was cut into 1 mm × 1 mm cubes and destained three times by being washed with 100 μL of 100 mM ammonium bicarbonate for 15 min and 100 μL of acetonitrile for 15 min. The samples were dried in a speedvac. To digest the protein, the dried gel pieces were covered with ice-cold trypsin (0.01 μg/μL) in 50 mM ammonium bicarbonate for 30 min. After this time, the trypsin solution was removed and replaced with fresh 50 mM ammonium bicarbonate. The solution was incubated overnight at 37 °C. The peptides were extracted twice by the addition of 50 μL of a 0.2% formic acid/95% water/5% acetonitrile solution and vortex mixing at room temperature for 30 min. The supernatant was removed and saved. An additional 50 μL of a 0.2% formic acid/95% water/5% acetonitrile solution was added to the sample, and the mixture vortexed again at room temperature for 30 min. The supernatant was removed and combined with the supernatant from the first extraction. The combined trypsin-digested peptides were analyzed by UPLC coupled with LC-MS/MS using nanospray ionization. The nanospray ionization experiments were performed using an Orbitrap fusion Lumos hybrid mass spectrometer (Thermo) interfaced with a nanoscale, reversed phase UPLC system (Thermo Dionex UltiMate 3000 RSLC nano System) using a 25 cm, 75 μm (inside diameter) glass capillary packed with 1.7 μm C18 (130) BEH beads (Waters Corp.). Peptides were eluted from the C18 column into the mass spectrometer using a linear gradient (5% to 80%) of acetonitrile at a flow rate of 375 μL/min for 1 h. The following buffers were used to create the acetonitrile gradient: buffer A (98% H_2_O, 2% acetonitrile, and 0.1% formic acid) and buffer B (100% acetonitrile and 0.1% formic acid). The mass spectrometer parameters were as follows: mass range (*m*/*z*) of 400–1500 (using quadrupole isolation), 120 000 resolution setting, spray voltage of 2200 V, ion transfer tube temperature of 275 °C, AGC target of 400 000, and maximum injection time of 50 ms. Data-dependent scans were performed at top speed for most intense ions with the charge state set to include only +2–5 ions and a 5 s exclusion time, while selecting ions with minimal intensities of 50 000 at which the collision event was carried out in the high-energy collision cell (HCD collision energy of 30%), and the fragment masses were analyzed in the ion trap mass analyzer with an ion trap scan rate of turbo, a first mass *m*/*z* of 100, AGC Target 5000, and a maximum injection time of 35 ms. Protein identification and peptide identification were carried out using Byonic (Protein Metrics Inc.).

#### Coordinate Covalent Binding-Inductively Coupled Plasma Mass Spectrometry

Protein samples (50 μg) were incubated with the metal complex [50 μM, 2% (v/v) DMSO] in 200 μL of buffer for 2 or 6 h at room temperature with slow shaking in the dark. After this time, the protein/inhibitor mixture was placed in a Pierce protein PES concentrator (0.1–0.5 mL) with a molecular weight cutoff of 10 kDa. The solution was centrifuged at 10 000 rpm for 10 min. The concentrated protein was mixed with 0.5 mL of trace metal free water. The mixture was washed five times with trace metal free water. After this procedure, the protein was digested in concentrated trace metal free nitric acid. Each sample was diluted to a final volume of 1 mL with trace metal free water to a 5% aqueous nitric acid solution. The metal content of the sample was determined using an iCAP RQ inductively coupled plasma mass spectrometer (ICP-MS) and compared with those of reference standards. The obtained data were analyzed with Qtegra analysis software.

#### Reversibility of Coordinate Covalent Binding-Inductively Coupled Plasma Mass Spectrometry

3CL^pro^ (50 μg) was incubated with the metal complex [50 μM, 2% (v/v) DMSO] in 200 μL of buffer for 6 h at room temperature with slow shaking in the dark. After this time, the protein/inhibitor mixture was placed in a Pierce protein PES concentrator (0.1–0.5 mL) with a molecular weight cutoff of 10 kDa. The solution was centrifuged at 10 000 rpm for 10 min. The concentrated protein was mixed with 0.5 mL of trace metal free water. The mixture was washed five times with trace metal free water. The obtained protein–metallofragment adduct was divided into two equivalent portions. While the first portion was incubated in water, the second portion was incubated with a mixture of *tert*-butoxycarbonyl-l-cysteine methyl ester (0.25 mM) and glutathione (2 mM) in 200 μL of water for 2 h at room temperature with slow shaking. After this time, the protein/inhibitor mixture was placed in a Pierce protein PES concentrator (0.1–0.5 mL) with a molecular weight cutoff of 10 kDa. The solution was centrifuged at 10 000 rpm for 10 min. The concentrated protein was mixed with 0.5 mL of trace metal free water. The mixture was washed five times with trace metal free water. After this procedure, the first and second portions of the protein were digested in concentrated trace metal free nitric acid. Each sample was diluted to a final volume of 1 mL with trace metal free water to a 5% aqueous nitric acid solution. The metal content of the sample was determined using an iCAP RQ inductively coupled plasma mass spectrometer (ICP-MS) and compared with those of reference standards. The obtained data were analyzed with Qtegra analysis software.

#### Computationally Predicted Binding Poses

The geometry of a metal complex was determined using density functional theory calculations with the Gaussian software package (Gaussian, Inc., Wallingford, CT). The metal atom was described using the Los Alamos (LANL2) effective core potential with the corresponding triple-ζ basis set while all other atoms were described with the Pople double-ζ basis set with a single set of polarization functions on non-hydrogen atoms [6-31G(d)]. Solvent effects were included using a polarizable continuum model (PCM). The structures of all calculated molecules correspond to ground state minima on the ground state potential energy surfaces with no imaginary frequencies present. The geometry of the calculated structures was verified by comparison with those of the crystal structures of structurally related compounds from the CCDC. The aqua molecule, which was placed as a dummy molecule for the metal–cysteine interaction, was removed, and the obtained structure fixed. The structures of 3CL^pro^ (PDB entry 6Y2F), PL^pro^ (PDB entry 7NFV), CatB (PDB entry 6AY2), and CatL (PDB entry 3HHA) were prepared using the molecular operating environment (MOE, Chemical Computing Group ULC, Montreal, QC) software package by removal of the bound ligand and water molecules and protonation. The metal complex fragment was covalently docked toward all thiol residues found in the protein. The generated docking poses were energetically minimized using the GBVI/WSA dG force fields in MOE.

#### 3CL^pro^ Enzymatic Assay

A slightly modified protocol from the commercially available assay (BPS Bioscience) was used. Dithiothreitol was substituted with tris(2-carboxyethyl)phosphine (TCEP), the latter of which was found not to alter the activity of the enzyme in the assay. The 3CL^pro^ protease was thawed on ice and activated by dilution to 10.0 ng/μL with assay buffer. The enzyme solution was further diluted with assay buffer to 0.5 ng/μL. Twenty microliters of the enzyme solution was mixed with 5 μL of increasing concentrations of the complex [2% (v/v) DMSO] diluted in assay buffer in the dark. The mixture was incubated for 30 min at 37 °C with slow shaking. The substrate [Dabcyl-KTSAVLQSGFRKM-E(Edans)-NH_2_] was diluted to 50 μM, and 25 μL was added to the enzyme mixture. The mixture was incubated for 4 h at 37 °C with slow shaking. The generated fluorescence signal (λ_ex_ = 360 nm; λ_em_ = 460 nm) was recorded with a Synergy H4 (BioTek) microplate reader. As a positive control, the known inhibitor GC376 (IC_50_ = 140 ± 20 nM) was used.

#### PL^pro^ Enzymatic Assay

A slightly modified protocol from a previous publication^40^ was used. The PL^pro^ enzyme (Elabscience) was thawed on ice and activated by dilution to 10.0 ng/μL with HEPES buffer. The enzyme solution was further diluted with HEPES buffer to 0.5 ng/μL. Twenty microliters of the enzyme solution was mixed with 5 μL of increasing concentrations of the complex [2% (v/v) DMSO] diluted in assay buffer in the dark. The substrate (Z-Arg-Leu-Arg-Gly-Gly-AMC, Bachem Bioscience) was diluted to 10 μM, and 25 μL was added to the enzyme mixture. The mixture was incubated for 60 min at 37 °C with slow shaking. The generated fluorescence signal (λ_ex_ = 355 nm; λ_em_ = 460 nm) was recorded with a Synergy H4 (BioTek) microplate reader. As a positive control, the known inhibitor GRL-0617 (IC_50_ = 5 ± 2 μM) was used.

#### Cathepsin B Enzymatic Assay

A slightly modified protocol from the commercially available assay (BPS Bioscience) was used. Dithiothreitol was substituted with tris(2-carboxyethyl)phosphine (TCEP), the latter of which was found not to alter the activity of the enzyme in the assay. The cathepsin B enzyme was thawed on ice and activated by dilution to 10.0 ng/μL with assay buffer. The enzyme solution was further diluted with assay buffer to 0.02 ng/μL. Twenty microliters of the enzyme solution was mixed with 5 μL of increasing concentrations of the complex [2% (v/v) DMSO] diluted in assay buffer in the dark. The mixture was incubated for 10 min at 37 °C with slow shaking. The substrate (Z-Leu-Arg-AMC) was diluted to 10 μM, and 25 μL was added to the enzyme mixture. This yields a mixture containing 10 mM Tris-HCl, 0.05% glycerol, 300 μM TCEP, and 10 μM cathepsin B substrate. The mixture was incubated for 60 min at 37 °C with slow shaking. The generated fluorescence signal (λ_ex_ = 360 nm; λ_em_ = 460 nm) was recorded with a Synergy H4 (BioTek) microplate reader. As a positive control, the known inhibitor E-64 (IC_50_ = 4 ± 2 nM) was used.

#### Cathepsin L Enzymatic Assay

A slightly modified protocol from the commercially available assay (BPS Bioscience) was used. Dithiothreitol was substituted with tris(2-carboxyethyl)phosphine (TCEP), the latter of which was found not to alter the activity of the enzyme in the assay. The cathepsin L enzyme was thawed on ice and activated by dilution to 10.0 ng/μL with assay buffer. The enzyme solution was further diluted with assay buffer to 0.02 ng/μL. Twenty microliters of the enzyme solution was mixed with 5 μL of increasing concentrations of the complex [2% (v/v) DMSO] diluted in assay buffer in the dark. The substrate (Z-Leu-Arg-AMC) was diluted to 10 μM, and 25 μL was added to the enzyme mixture. This yields a mixture containing 10 mM Tris-HCl, 0.05% glycerol, 300 μM TCEP, and 10 μM cathepsin L substrate. The mixture was incubated for 60 min at 37 °C with slow shaking. The generated fluorescence signal (λ_ex_ = 360 nm; λ_em_ = 460 nm) was recorded with a Synergy H4 (BioTek) microplate reader. As a positive control, the known inhibitor E-64 (IC_50_ = 33 ± 9 nM) was used.

#### DPP4 Enzymatic Assay

A slightly modified protocol from the commercially available assay (BPS Bioscience) was used. The DPP4 enzyme was thawed on ice and diluted to 0.1 ng/μL with assay buffer, and the substrate (Ala-Pro-AMC dipeptide) diluted to 100 μM with assay buffer. Eighty microliters of the assay buffer was mixed with 5 μL of the substrate, 5 μL of increasing concentrations of the complex [2% (v/v) DMSO] diluted in assay buffer, and 10 μL of the DPP4 enzyme in the dark. This yields a mixture containing 10 mM Tris-HCl, 10 mM MgCl_2_, 0.05% Tween 20, and 20 μM DPP4 substrate at pH 7.4. The mixture was incubated for 60 min at 37 °C with slow shaking. The generated fluorescence signal (λ_ex_ = 360 nm; λ_em_ = 460 nm) was recorded with a Synergy H4 (BioTek) microplate reader. The difference in fluorescence signals was correlated to the concentration of the complex, and the IC_50_ values were determined. As a control substance, the well-known inhibitor sitagliptin (IC_50_ = 23 ± 9 nM) was used.

#### BACE1 Enzymatic Assay

A slightly modified protocol from the commercially available assay (BPS Bioscience) was used. The BACE1 enzyme was thawed on ice and diluted to 7.5 ng/μL with assay buffer. Then, 69 μL of the assay buffer was mixed with 1 μL of the FRET substrate, 10 μL of increasing concentrations of the complex [2% (v/v) DMSO] diluted in inhibitor buffer, and 20 μL of the BACE1 enzyme in the dark. This yields a mixture containing 10 mM NaOAc, HOAc, and BACE1 substrate at pH 7.4. The fluorescence signal (λ_ex_ = 320 nm; λ_em_ = 405 nm) was recorded with a Synergy H4 (BioTek) microplate reader. The plate was immediately covered with aluminum foil, kept in the dark, and incubated for 20 min at 37 °C with slow shaking. The generated fluorescence signal (λ_ex_ = 320 nm; λ_em_ = 405 nm) was recorded with a Synergy H4 (BioTek) microplate reader. The difference in fluorescence intensity was correlated to the concentration of the complex, and the IC_50_ values were determined. As a positive control, the known inhibitor verubecestat (IC_50_ = 37 ± 8 nM) was used.

#### Furin Enzymatic Assay

A slightly modified protocol from the commercially available assay (BPS Bioscience) was used. The furin enzyme was thawed on ice and activated by dilution to 10.0 ng/μL with assay buffer. The enzyme solution was further diluted with assay buffer to 0.5 ng/μL. Then, 50 μL of the enzyme solution was mixed with 10 μL of increasing concentrations of the complex [2% (v/v) DMSO] diluted in assay buffer in the dark. The substrate was diluted to 5 μM, and 40 μL was added to the enzyme mixture. The mixture was incubated for 30 min at 37 °C with slow shaking. The generated fluorescence signal (λ_ex_ = 380 nm; λ_em_ = 460 nm) was recorded with a Synergy H4 (BioTek) microplate reader. As a positive control, the known inhibitor chloromethylketone (IC_50_ = 4 ± 0.5 nM) was used.

#### Parallel Artificial Membrane Permeability Assay

The cell permeability of the metal complexes was assessed using an artificial membrane using a slight modification to a reported protocol.^57^ The wells in the acceptor plate were filled with a phosphate-buffered saline solution (300 μL). Then, 4% lecithin in dodecane (5 μL) was carefully placed on top of the membrane of the donor plate. The complex [200 μL, 100 μM, 2% (v/v) DMSO] diluted in water was added to the wells of the donor plate. The tray of the donor plate was placed inside the acceptor plate. The combined plates were incubated for 8 h at room temperature in the dark. The donor plate was removed, and the absorbance at 350 nm in each well in the acceptor plate determined. As control substances, commercially supplied reference compounds with high (0.055 ± 0.005 μm/s), medium (0.028 ± 0.004 μm/s), and low (0.009 ± 0.002 μm/s) cell permeabilities were used. Using the following equation, the permeability rate of the compound was determined:
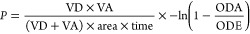
where *P* is the permeability rate in centimeters per second, VD is the donor volume (0.2 cm^3^), VA is the acceptor volume (0.3 cm^3^), area is 0.24 cm^2^, time is the incubation time in seconds, ODA is the absorbance of the acceptor solution, and ODE is the absorbance of the equilibrated acceptor solution.

#### Preparation of the Mouse Proteome

Mouse tissue (heart, lung, kidney, intestine, and liver) was harvested from 4-week-old C57Bl6/N female mice and provided by the animal core facility located at the University of California, San Diego. The tissue was immediately flash frozen in liquid nitrogen for storage. The different types of tissues from a single animal model were combined and treated with lysis buffer (150 mL containing 1.5 mL of Triton-X, 26.1 mg of dithiothreitol, 2 mg of DNase-1, 150 mg of lysozyme, 7.5 mL of glycerol, and 2 tablets of protease inhibitor). The suspension was treated with an ultrasonic pulse program (pulse of 20 s, break of 59 s, power of 60%, and combined treatment time of 20 min) using a FB120 probe sonicator (Fisher Scientific). The sample was centrifuged at 4500 rpm and 4 °C for 30 min. The supernatant solution was collected. The pellet was resuspended in lysis buffer (50 mL), and the suspension was treated with an ultrasonic pulse program (pulse of 20 s, break of 59 s, power of 60%, and combined treatment time of 40 min) using a model FB120 probe sonicator (Fisher Scientific). The prior supernatant solution and the solution obtained upon further treatment of the pellet were combined. The lysis buffer was exchanged with phosphate-buffered saline (PBS) by dialysis (MWCO of 10 000) at 4 °C overnight. The protein concentration of the sample was determined with a Pierce bicinchoninic acid protein assay kit (Fisher Scientific). Aliquots of the solution were prepared and stored at −80 °C.

#### Identification of Labeled Proteins within the Mouse Proteome

The performance of compound **14** was compared to that of the reported acrylate alkyne warhead oct-1-en-7-yn-3-one.^58^ The prepared mouse proteome (2 μL, 23.7 μg/μL) was diluted with water (10 μL), and the mixture incubated with the warhead (1 μL, 200 μM) at room temperature for 2 h. After this time, the solution was incubated with rhodamine 110-azide (1 μL, 200 μM), copper(II) sulfate (1 μL, 25 mM), and sodium ascorbate (1 μL, 25 mM) for 2 h. Each solution was further incubated with a nonreducing, fluorescent compatible sample buffer (5 μL, Fisher Scientific). The mixture was heated at 98 °C for 5 min and then allowed to cool to room temperature. As a reference for the bands, the Precision Plus Protein Unstained Protein Standard Ladder (Bio-Rad) was used. The prepared samples were then separated by 4% to 20% mini-PROTEAN TGX stain free SDS–PAGE (Bio-Rad) analysis. The gel was rinsed with water, and the bands were visualized with an Amersham Imager 680 instrument (λ_ex_ = 492 nm; λ_em_ = 508 nm). To verify the presence of the proteome, the gel was stained with a Coomassie Brilliant Blue R-250 solution (Bio-Rad). The gel was washed three times with water, and the bands were visualized with an Amersham Imager 680 instrument. Final concentrations within this assay are 2.37 μg/μL proteome, 10 μM warhead, 10 μM rhodamine dye, 625 μM copper(II) sulfate, and 625 μM sodium ascorbate.

## Supporting Information Available

Synthetic schemes, HPLC traces of compounds, images of docking poses, proteome gel stains, inhibition curves, and additional mass spectrometry data (PDF)
Molecular formula strings (CSV)

## Abbreviations

CatB: cathepsin B
CatL: cathepsin L
3CL^pro^: 3-chymotrypsin-like protease
DMSO: dimethyl sulfoxide
IC_50_: half-maximal inhibitory concentration
ICP-MS: inductively coupled plasma mass spectrometer
PL^pro^: papain-like protease
PBS: phosphate-buffered saline
PDB: Protein Data Bank
rt: room temperature
SDS–PAGE: sodium dodecyl sulfate–polyacrylamide gel electrophoresis
TCEP: tris(2-carboxyethyl)phosphine
