# Correction to “Rhenium(V) Complexes as Cysteine-Targeting Coordinate Covalent Warheads”

**DOI:** 10.1021/acs.jmedchem.5c02640

**Published:** 2025-09-29

**Authors:** Johannes Karges, Seth M. Cohen

We note that a figure associated with data discussed in the main manuscript was missing and would like to use this correction to report this missing figure.

In addition, the main text of our published manuscript had incorrect figure referencing that we correct here. The text marked in **bold underlined** highlight refers to the correct figure referencing. The remainder of the text is unchanged.

The human enzyme CatB did not efficiently ionize under the ESI-MS conditions used, and as such, it was not possible to investigate the binding of **3** by ESI-MS. However, binding of **3** to CatB was studied by ICP-MS. Upon incubation of **3** for 2 or 6 h, an average of 1.3 ± 0.2 or 1.8 ± 0.3 equiv of Re was found bound to CatB (Figure 5 **a**). CatB possesses 14 cysteine residues (Cys14, Cys26, Cys29, Cys43, Cys62, Cys63, Cys67, Cys71, Cys100, Cys108, Cys119, Cys128, Cys132, and Cys240). Despite the large number of cysteine residues in CatB, the majority of these form disulfide bonds or are in the interior of the protein. Only residues Cys29 and Cys240 can be accessed from the surface and could present sites of adduct formation. This is consistent with the ICP-MS data, and docking experiments were used to show the adducts of **3** bound to these two residues (Figure 5 **b**). As the compound was predicted to interact with the catalytic Cys29 residue, it is expected that the metal complex should be able to inhibit the activity of CatB.

**Figure 5:**
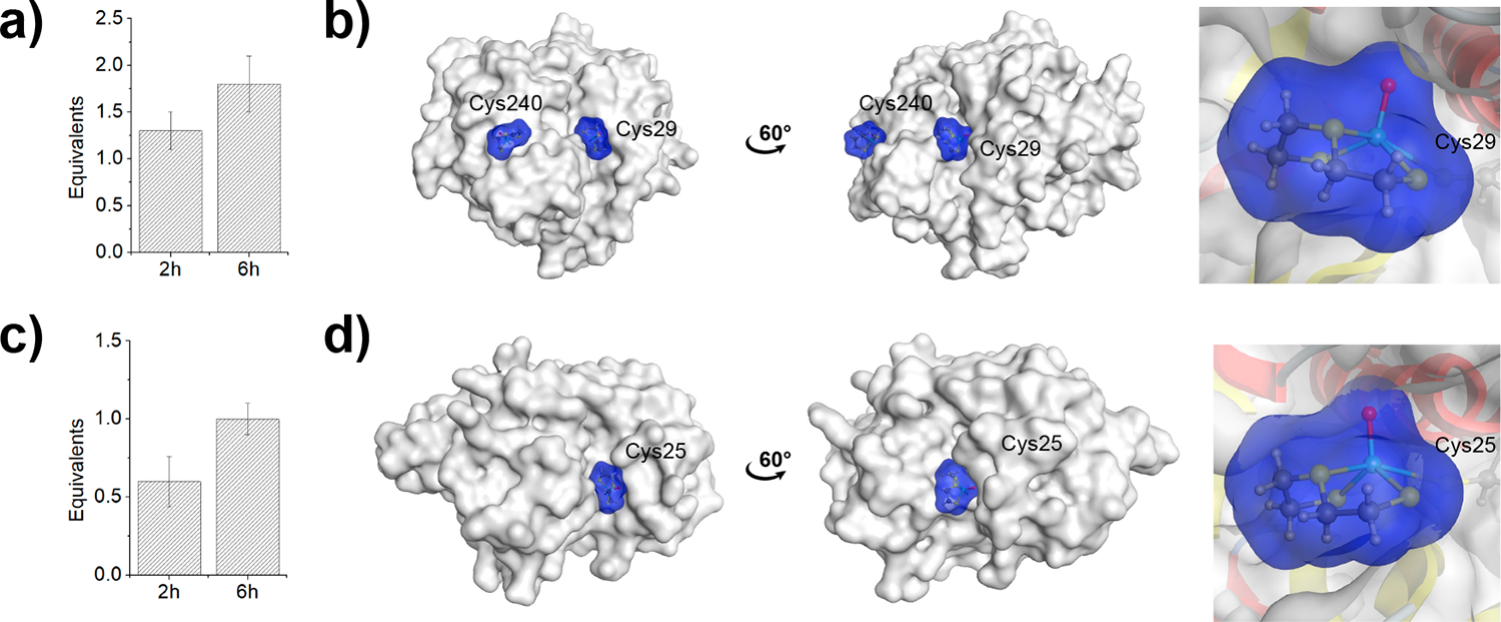
Coordinative covalent binding of metallofragment **3** to CatB and CatL. a) Determination of equivalents of Re bound to CatB via ICP-MS analysis after co-incubation with the Re metallofragment for 2 or 6 h. b) Computationally determined binding poses of the metallofragment with cysteine residues in CatB (PDB: 6AY2) visualized using a protein surface representation. Zoomed in image of the binding pose with the catalytically active Cys29 residue of CatB. c) Determination of equivalents of Re bound to CatL via ICP-MS analysis after co-incubation with the Re metallofragment for 2 or 6 h. d) Computationally determined binding poses of the metallofragment with cysteine residues in CatL (PDB: 3HHA) visualized using a protein surface representation. Zoomed in image of the binding pose with the catalytically active Cys25 residue of CatL.

In a similar manner, the binding of **3** to human CatL was studied by ICP-MS. The analysis of the metal content revealed that upon incubation of **3** with CatL for 2 and 6 h an average of 0.6 ± 0.2 and 1.0 ± 0.1 equiv of Re were coordinated to the protein (Figure 5 **c**). CatL possesses seven cysteine residues (Cys22, Cys25, Cys56, Cys65, Cys98, Cys156, and Cys209), but the structure shows that only Cys25 is located on the surface. Computational docking experiments confirm that a coordinate covalent interaction with **3** was formed with only Cys25 (Figure 5 **d**). As Cys25 is the key catalytic residue, it is predicted that **3** should inhibit CatL.

The authors sincerely apologize for these oversights and errors.

