# Supplementary material for: Rhenium(V) Complexes as Cysteine-Targeting Coordinate Covalent Warheads
Source: J Med Chem. 2023 Feb 8;66(4):3088–105. doi: 10.1021/acs.jmedchem.2c02074 (PMC9969397; doi:10.1021/acs.jmedchem.2c02074)
Supplement: Supplementary file 1 — jm2c02074_si_001.pdf [file jm2c02074_si_001.pdf]

Supporting Information

Rhenium(V) Complexes as Cysteine Targeting Coordinate Covalent Warheads

*Johannes Karges and Seth M. Cohen**

Department of Chemistry and Biochemistry, University of California, San Diego, La Jolla, California 92093, United States.

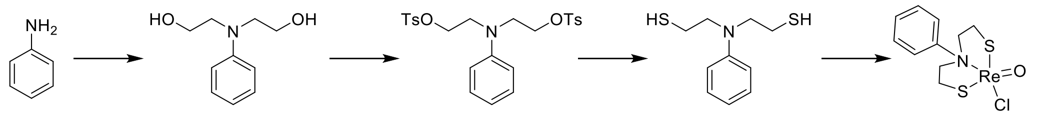

Scheme S1. Synthesis of ReO(*N*,*N*-Bis(2-thioethyl)aniline)(chloride) (**9**).

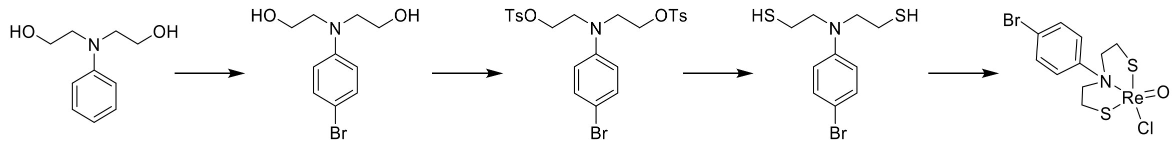

Scheme S2. Synthesis of ReO(*N*,*N*-Bis(2-thioethyl)-4-bromoaniline)(chloride) (**10**).

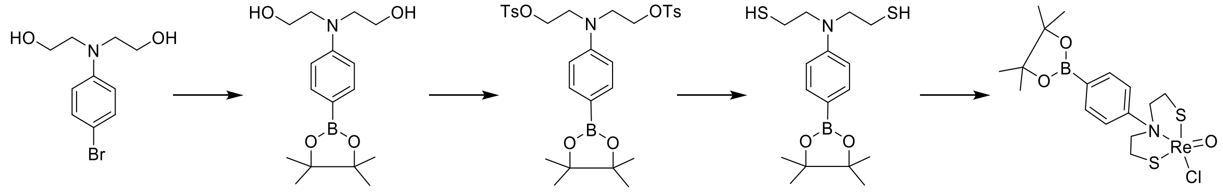

Scheme S3. Synthesis of ReO(*N*,*N*-Bis(2-thioethyl)-4-boronic acid pinacol ester aniline)(chloride) (**11**).

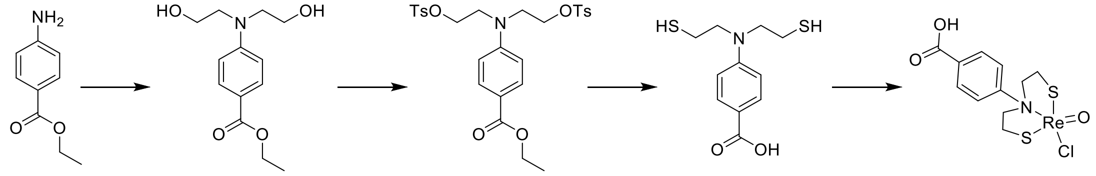

Scheme S4. Synthesis of ReO(*N*,*N*-Bis(2-thioethyl)-4-carboxlic acid aniline)(chloride) (**12**).

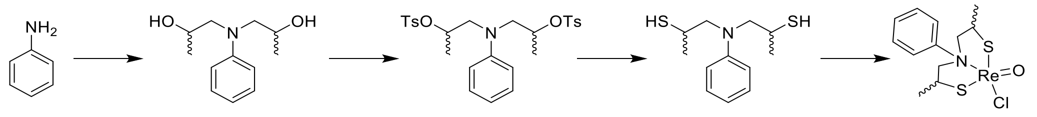

Scheme S5. Synthesis of ReO(*N*,*N*-Bis(2-thiopropyl)aniline)(chloride) (**13**).

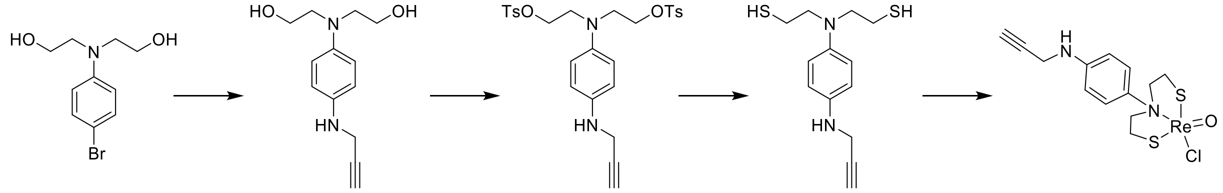

Scheme S6. Synthesis of ReO(*N*,*N*-Bis(2-thioethyl)-4-(Prop-2-yn-1-yloxy)aniline)(chloride) (**14**).

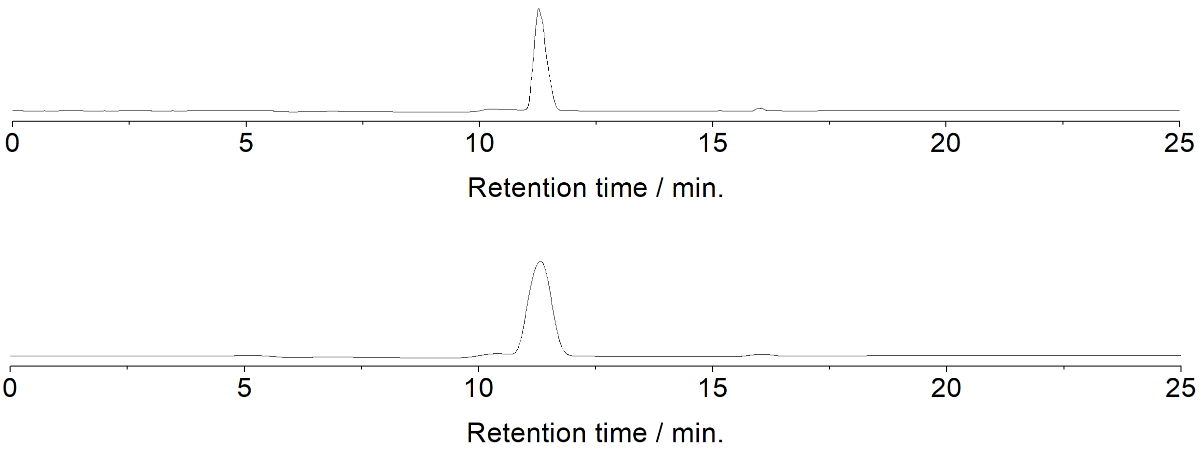

Figure S1. HPLC chromatogram at 250 nm of **3** upon incubation at 0 h (top) and after 48 h (bottom) in phosphate-buffered saline.

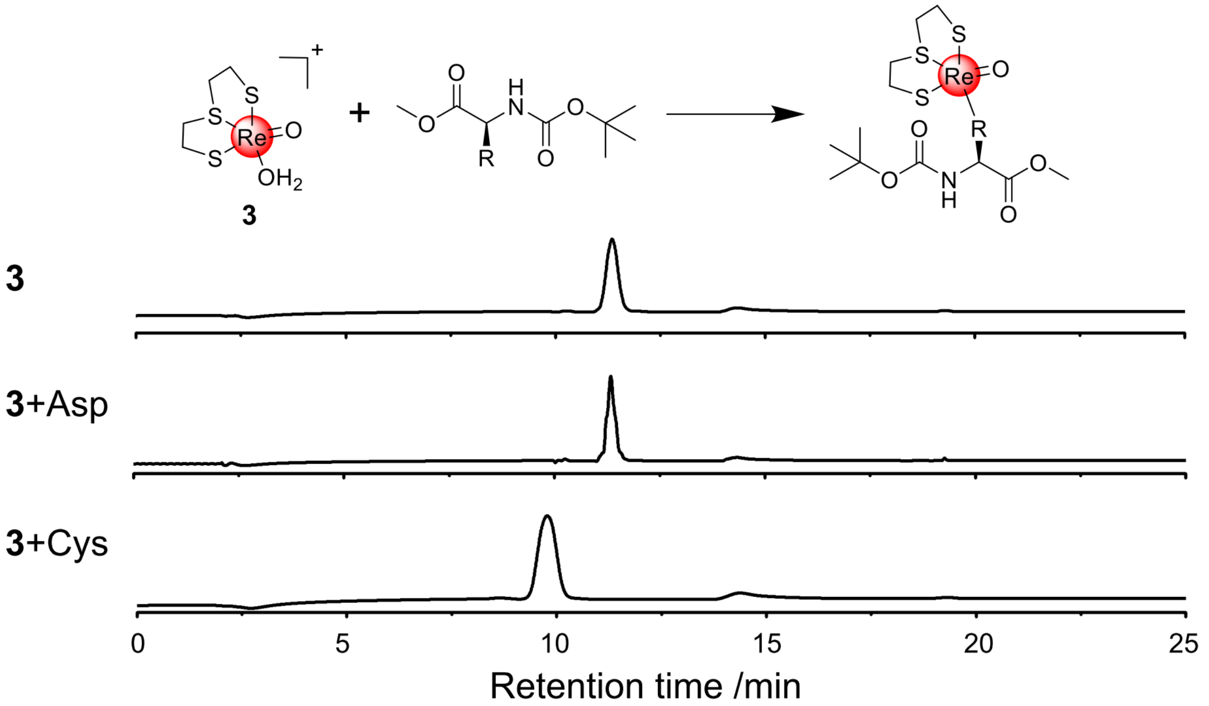

Figure S2. HPLC traces upon incubation of the Re(V) complex **3** (1 mg/mL) with Asp or Cys in water at 37 °C for 4 h in the dark.

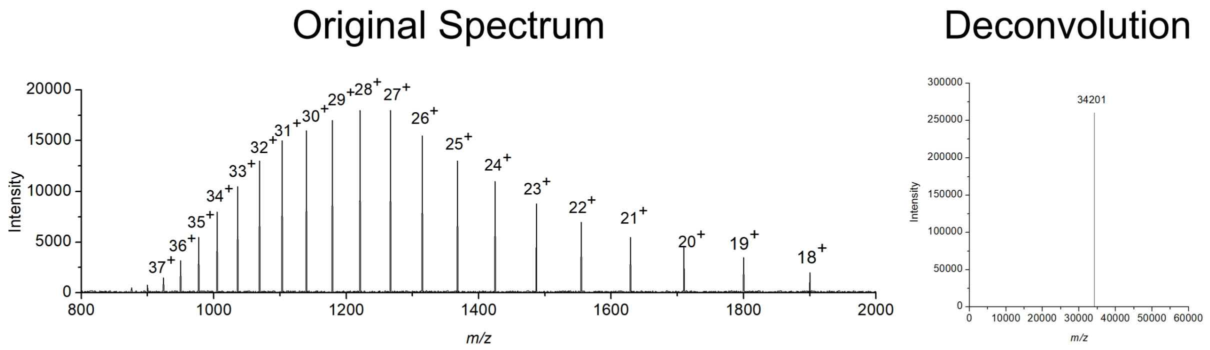

Figure S3. Original and deconvoluted mass spectrum of the product of organic covalent inhibitor GC376 and 3CL^{pro}.

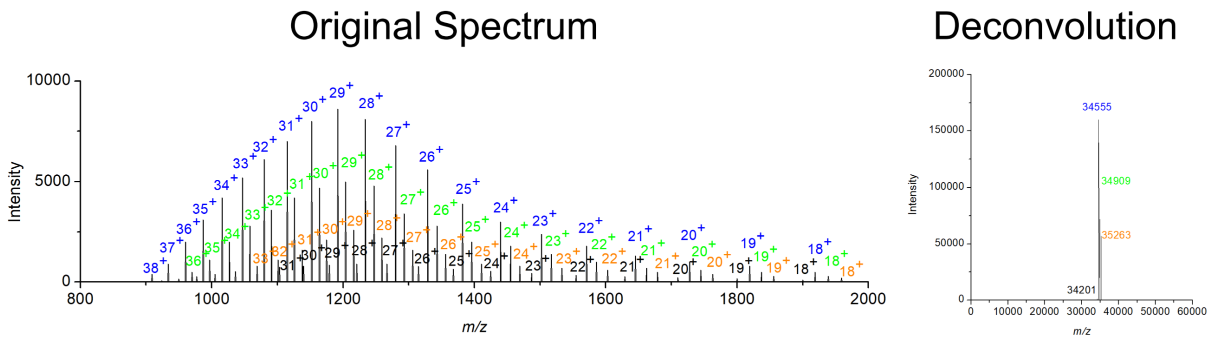

Figure S4. Original and deconvoluted mass spectrum of the coordinative covalent binding of the 3CL^{pro}-GC376 adduct with **3**. The spectrum shows a distribution of the coordinative covalent binding of GC376 (black), one (blue), two (green), and three (orange) Re(V) complexes to 3CL^{pro}.

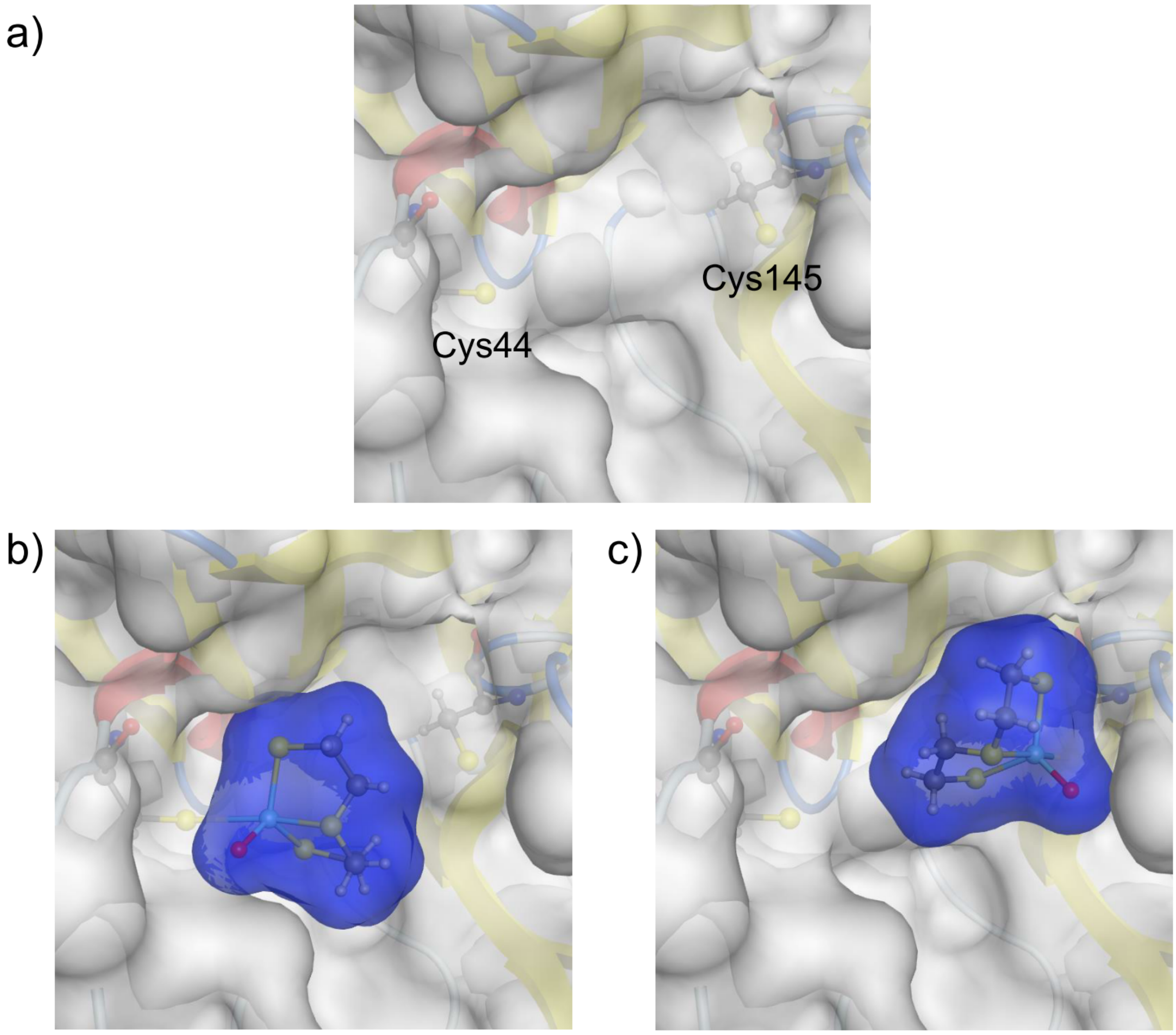

Figure S5. a) Highlighting of Cys44 and Cys145 in the active site of $3CL^{pro}$ visualized by protein surface representations. b) Possible binding pose of the Re(V) metallofragment **3** with Cys44 in $3CL^{pro}$ visualized by protein surface representations. c) Possible binding pose of the Re(V) metallofragment **3** with Cys145 in $3CL^{pro}$ visualized by protein surface representations. PDB: 6Y2F.

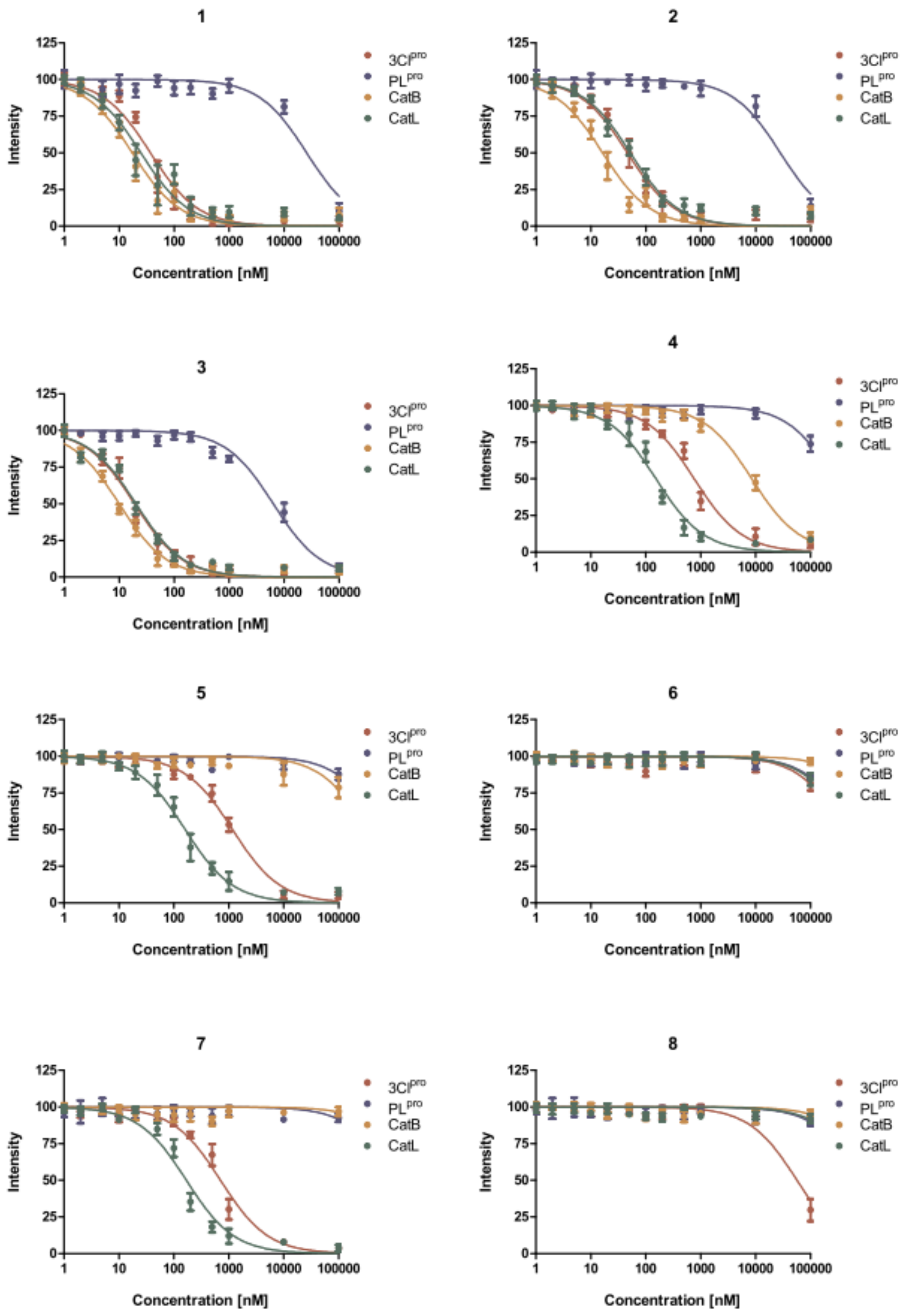

Figure S6. Dose-response curves of 3CL^{pro} (red), PL^{pro} (blue), CatB (orange), and CatL (green) with metallofragments **1-8**.

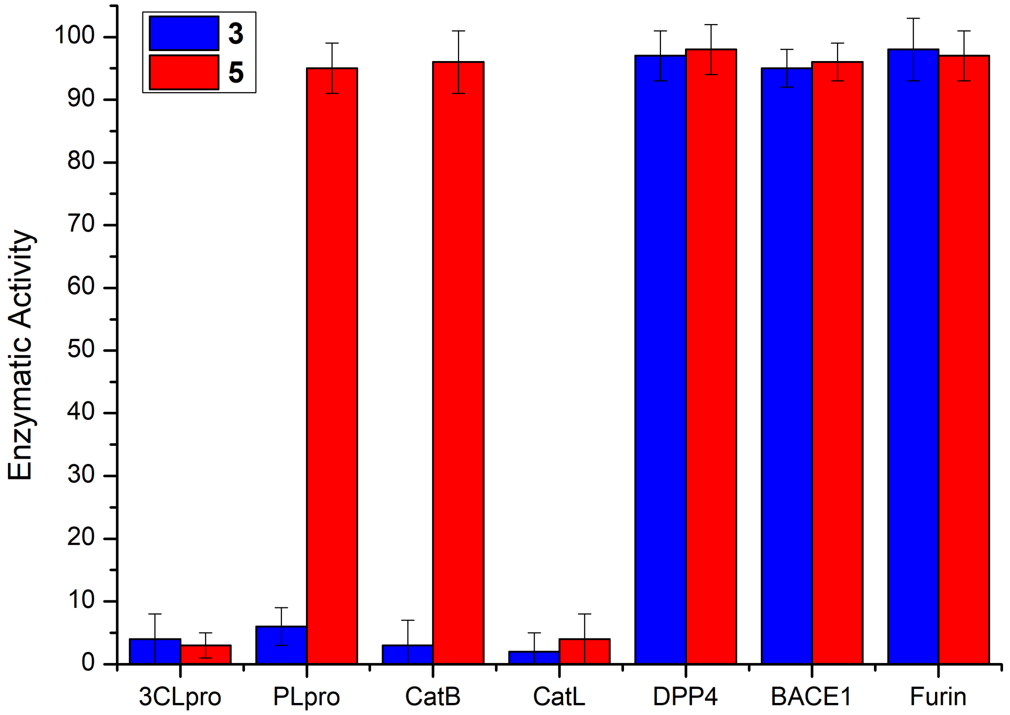

Figure S7. Inhibitory activity of compounds **3** and **5** (concentration of 50 µM) towards human serine protease dipeptidyl peptidase-4 (DPP4), aspartate protease beta-secretase 1 (BACE1), and serine protease Furin in comparison to SARS-CoV-2 associated cysteine proteases 3CL^{pro}, PL^{pro}, CatB, and CatL.

Table S1. Cysteine residues human serine protease dipeptidyl peptidase-4 (DPP4), aspartate protease beta-secretase 1 (BACE1), and serine protease Furin.

Enzyme	PDB-Code	Cysteine Residues	Surface Accessible Cysteine Residues
DPP4	5T4E	Cys301, Cys328, Cys339, Cys385, Cys394, Cys444, Cys447, Cys454, Cys472, Cys551, Cys649, Cys762	Cys339, Cys385, Cys394, Cys444, Cys447
BACE1	4IVT	Cys155, Cys217, Cys269, Cys319, Cys359, Cys382	Cys155, Cys217, Cys269, Cys319, Cys359, Cys382
Furin	7O1U	Cys198, Cys211, Cys303, Cys305, Cys333, Cys360, Cys450, Cys474	Cys211, Cys360, Cys450, Cys474

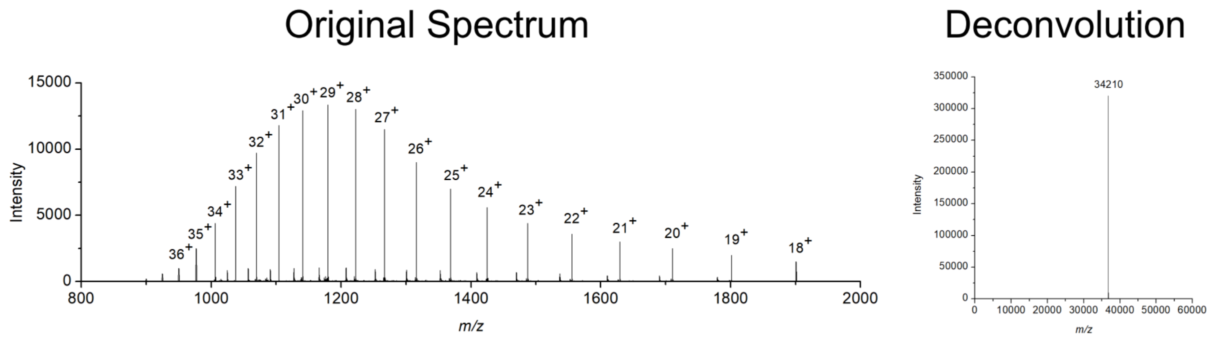

Figure S8. Original and deconvoluted mass spectrum of the Re(V) metallofragment **9** and 3CL^{pro}.

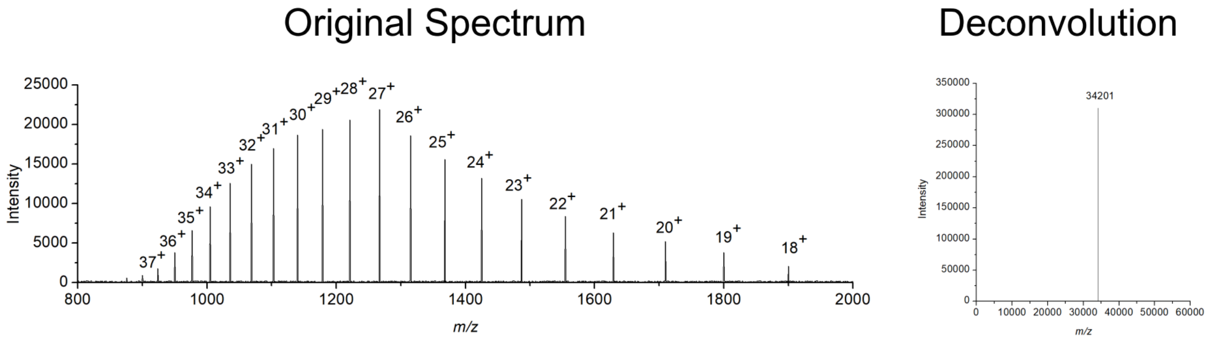

Figure S9. Original and deconvoluted mass spectrum of the incubation of the 3CL^{pro}-GC376 adduct with **9**. The spectrum shows only 3CL^{pro}-GC376.

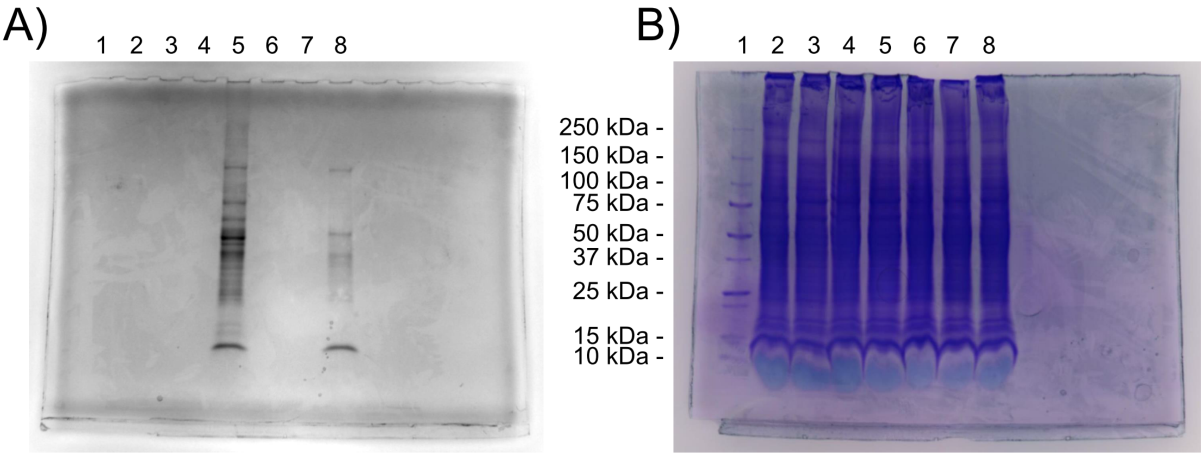

Figure S10. (A) Fluorescence (λ_{ex} = 492 nm, λ_{em} = 508 nm) or (B) Coomassie staining of a sodium dodecyl sulfate–polyacrylamide gel electrophoresis (SDS-PAGE) gel of a mouse proteosome incubated with the Re(V) alkyne warhead **14** or the literature known acrylate alkyne warhead oct-1-en-7-yn-3-one (E. Weerapana, G. M. Simon, B. F. Cravatt *Nat. Chem. Bio.* **2008**, *4*, 405-407) under various conditions. Lanes 1: MW Ladder, 2: DMSO, 3: oct-1-en-7-yn-3-one waread, 4: rhodamine dye, 5: oct-1-en-7-yn-3-one + rhodamine dye + copper(II) sulfate + sodium ascorbate, 6: compound **14**, 7: rhodamine dye, 8: compound **14** + rhodamine dye + copper(II) sulfate + sodium ascorbate. Final concentrations within this assay are: proteome (2.37 µg/µL), oct-1-en-7-yn-3-one or **14** (10 µM), rhodamine dye (10 µM), copper(II) sulfate (625 µM), and sodium ascorbate (625 µM).

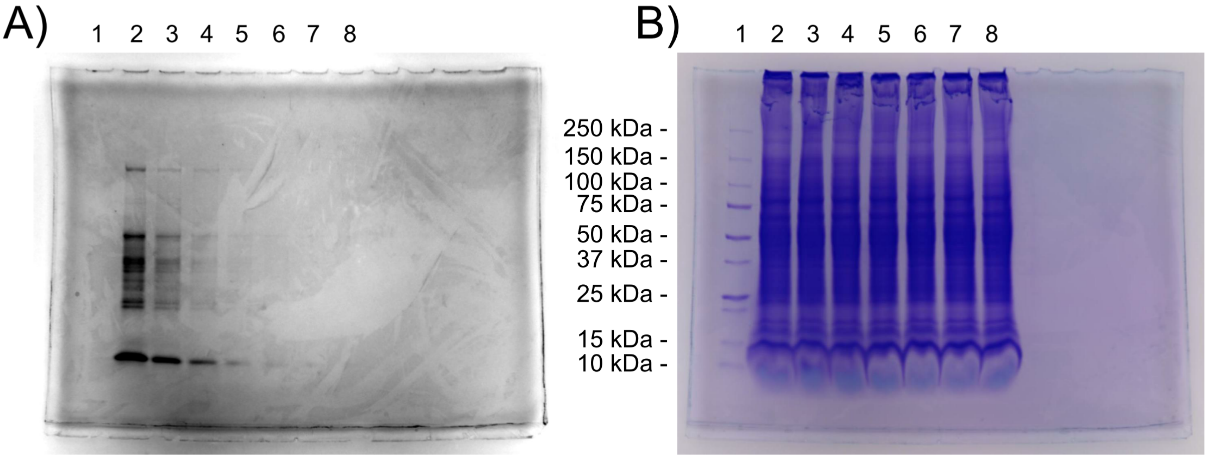

Figure S11. (A) Fluorescence (λ_{ex} = 492 nm, λ_{em} = 508 nm) or (B) Coomassie staining of a sodium dodecyl sulfate–polyacrylamide gel electrophoresis (SDS-PAGE) gel of a mouse proteosome incubated with decreasing concentrations of the Re(V) alkyne warhead **14**. Lanes 1: Ladder, 2: 50 μM, 3: 20 μM, 4: 10 μM, 5: 5 μM, 6: 2.5 μM, 7: 1 μM, 8: 0.1 μM. Final concentrations within this assay are: proteome (2.37 μg/μL), rhodamine dye (10 μM), copper(II) sulfate (625 μM), and sodium ascorbate (625 μM).
